# Solvent Isotope Effect on the Thermodynamics of Complexation with Cyclodextrins and Cucurbiturils: An ITC Study in H_2_O and D_2_O

**DOI:** 10.3390/molecules31193483

**Published:** 2026-09-29

**Authors:** Josip Požar, Marija Alešković, Marina Šekutor, Vladislav Tomišić, Andrea Usenik

**Affiliations:** 1 Department of Chemistry, University of Zagreb Faculty of Science, Horvatovac 102A, HR-10000 Zagreb, Croatia; pozar@chem.pmf.hr (J.P.); vtomisic@chem.pmf.hr (V.T.); 2 Department of Organic Chemistry and Biochemistry, Ruđer Bošković Institute, Bijenička 54, HR-10000 Zagreb, Croatia; marija.aleskovic@irb.hr (M.A.); msekutor@irb.hr (M.Š.)

**Keywords:** heavy water, NMR spectroscopy, solvent isotope effect, isothermal titration calorimetry, cyclodextrins, cucurbiturils, hydrophobic effect

## Abstract

Determination of supramolecular host–guest complex stability constants often relies on ^1^H NMR spectroscopy, particularly when it comes to reactants lacking chromophores. This is usually the case for hydrophobically driven complexation, where hosts such as cyclodextrins and cucurbiturils interact with small, scarcely soluble guests in aqueous media. Even sophisticated solvent suppression techniques still require a small percentage of deuterated water, so the reactions are usually affected by the solvent isotope effect, either kinetically or thermodynamically, the latter being significantly less investigated. In this work, we report a systematic calorimetric study of the influence of solvent deuteration on the thermodynamic reaction parameters (Δ_r_*X*°, where *X* = *G*, *H*, *S*, *C_p_*) in 278−338 K temperature range for the reactions involving cyclodextrins and cucurbiturils and two hydrophobic alcohols. In accord with previous systematic investigations carried out solely at 298 K, the complex stability constants did not vary significantly when transferred from H_2_O to D_2_O. This was, in the case of cyclodextrins, a consequence of a partial enthalpy–entropy compensation, as the differences between Δ_r_*H*° and –*T*Δ_r_*S*° in two media were similar in magnitude but opposite in sign (up to 5 kJ mol^−1^, excluding one outlier). Thus, our finding indicates that differences in the hydration of the hosts and guests in regular and heavy water partially compensate for each other.

## 1. Introduction

The first sample of pure deuterium oxide (D_2_O), commonly known as heavy water, was isolated in 1933 [1,2], two years after the discovery of deuterium [3] and one year after water enriched in D_2_O was first obtained by fractional electrolysis [4]. Ever since, heavy water has intrigued physicists and chemists alike.

For many years, D_2_O has played a crucial role in nuclear energy production and, unfortunately, also became strategically important in wartime nuclear programs. Research into the chemical properties of H_2_O and D_2_O began in the following year [5,6], with the first studies of heavy water’s physical properties carried out in parallel [2,7]. As expected, D_2_O was found to be roughly 10% denser than water with natural isotopic abundance. Its molar heat capacity was also higher than that of H_2_O [8,9,10,11,12], as well as its normal boiling and freezing points [9,13,14], suggesting stronger hydrogen bonding in D_2_O than in H_2_O. Interestingly, although the electronic contribution alone would result in approximately 1% weaker O–D···O bond compared to its protium analog [15], nuclear quantum effects act in the opposite direction, rendering the deuterium hydrogen bond approximately 0.8−1.3 kJ mol^−1^ stronger than that in H_2_O [16,17,18,19]. Additionally, D_2_O forms more hydrogen bonds than its counterpart (3.76 on average compared to 3.62 in H_2_O) [20].

The pronounced influence of replacing protium with deuterium on the reaction rates [5,21,22,23] subsequently prompted extensive kinetic investigations of reactions in D_2_O. A particularly strong effect of isotope substitution was observed in enzyme-catalyzed metabolic reactions [24,25], with D_2_O found to be highly toxic to eukaryotic organisms [25]. Although much of the catalytic retardation arises from the mentioned primary kinetic isotope effect associated with proton-transfer [26,27], this is not the sole factor underlying reduced enzyme activity. Namely, stronger interactions with D_2_O can also impair catalytic activity of certain enzymes [28,29]. On the other hand, D_2_O has quite a limited effect on reaction rates of simpler supramolecular processes, such as crown ether-cation complexation reactions [30].

The thermodynamic reaction quantities also change upon replacing protium with deuterium, especially for cases where a proton is being transferred from one species to another. Namely, the autoprotolysis constant of D_2_O is lower than that of H_2_O (p*K*_w_ (298.15 K) = 14.87 and 13.995, respectively) [31,32], whereas the p*K*_a_ values of various acids typically increase by 0.4–0.6 units upon their transfer to D_2_O [6,33,34,35,36,37,38]. Solvent isotope substitution generally has a smaller effect on equilibrium constants (hence also on Δ_r_*G*°) of supramolecular reactions such as ligand:ion-pair formation or inclusion of guests inside cyclodextrins [39,40,41,42,43,44]. The solvent isotopic composition is thus routinely treated as a technical detail rather than as a thermodynamic variable. Deuterated water, for example, is widely used as a standard medium for studying biomolecular structure and dynamics, both because it reduces the dominant H_2_O signal in ^1^H NMR and because it provides useful contrast in neutron scattering [18,45]. Further, nuclear magnetic resonance is routinely used to determine drug–cyclodextrin complex stability constants in D_2_O [46,47,48,49], yet these values are subsequently applied to formulations in H_2_O. Similarly, dissociation constants of protein-ligand complexes obtained from NMR measurements in D_2_O buffers are commonly reported alongside calorimetric and spectroscopic values determined in H_2_O [50].

However, the documented comparative studies, on which this practice relies, were often limited to stability constant determinations, leaving open the possibility that similar Δ_r_*G*° values in D_2_O and water of natural hydrogen isotopic abundance arise from enthalpy–entropy compensation (Δ_r_*G*° = Δ_r_*H*° − *T*Δ_r_*S*°). Given the established differences in hydrogen-bond strength between D_2_O and H_2_O [16,17,18,19,20] this possibility warrants further consideration, especially in a wider temperature range, as both heat capacities and viscosities of D_2_O and H_2_O exhibit a pronounced temperature dependence [17,51,52].

The recent availability of sensitive and affordable isothermal titration calorimeters has enabled detailed thermodynamic investigations of the supramolecular processes taking place in D_2_O and H_2_O. For comparative thermodynamic studies in heavy water and that with natural isotope abundance, reactions that are particularly sensitive to reactant and product solvation are the most relevant. These undoubtedly include the binding of small molecules to proteins and the protein–protein association. Surprisingly, the literature data concerning the isotope effect on the corresponding reaction thermodynamics are extremely scarce. Connelly et al. investigated the binding of the hydrophobic macrolides tacrolimus (FK506) and rapamycin to FK506-binding protein FKBP-12 in H_2_O (pH = 7) and D_2_O (pD = 7) [53]. A large enthalpic destabilization in D_2_O, reaching up to 20 kJ mol^−1^ over 278–303 K was observed, which they attributed to differential hydration of protein and ligand atoms. Binding of both macrolides was also accompanied by large negative reaction heat capacities, consistent with burial of substantial nonpolar surface area of ligands upon complexation. Szalai et al. conducted ITC studies on the binding of five *p*-substituted benzamidines to trypsin and nine aromatic and heteroaromatic sulfonamides to bovine carbonic anhydrase II in both media, resulting in ΔΔ_r_*H*° values that ranged from −11.7 to 24.3 kJ mol^−1^, with only three ligands (sulfonamides) binding more exothermically in D_2_O, and the remaining eleven showing the opposite trend [54]. Despite considerable differences in Δ_r_*H*° in the examined solvents, a significantly smaller change in complex stability (ΔΔ_r_*G*° < 4.2 kJ mol^−1^ among the studied ligand series) was observed due to enthalpy–entropy compensation.

To the best of our knowledge, there are no comparative ITC studies of protein–protein binding in D_2_O and H_2_O; however, Eginton and Beckett examined the dimerization of the biotin repressor (BirA) in H_2_O and D_2_O using sedimentation equilibrium [55]. The association enthalpy, determined by van’t Hoff analysis (equilibrium constant temperature dependence assuming Δ_r_*C_p_*° ≈ 0) was far more favorable in D_2_O.

Because of particularly favorable hydration of ions, their binding with both synthetic and natural receptors should also be strongly affected by solvent isotope composition, especially for reactions resulting in extensive ion dehydration. The data are again surprisingly scarce. In contrast with endothermic transfer of calcium cation from H_2_O to D_2_O (Δ_t_*H* (Ca^2+^) = 5.4 kJ mol^−1^) [56], Carl et al. [57] reported that binding of Ca^2+^ to sodium polyacrylate in heavy water results in slightly more positive enthalpy changes (≈2 kJ mol^−1^). Similarly, the enthalpies of Rb^+^ hosting by a uranium cage-shaped macroion (U_60_) were found to be almost identical in H_2_O and D_2_O (≈0.5 kJ mol^−1^ difference) [58].

Finally, the association of hydrophobic species in water, commonly referred to as the hydrophobic effect, should be mentioned. These processes, which include micelle formation above CMC [59,60,61,62,63,64,65] and the inclusion of hydrophobic moieties inside the non- or weakly polar cavities of hosts such as cyclodextrins, cucurbiturils or cyclophanes [41,42,66,67,68,69], are almost completely solvent-driven and result in a release of quite a few water molecules into the bulk medium. As far as hydration of nonpolar functional groups and molecules in heavy water is concerned, their transfer to D_2_O is exothermic (Δ_t_*H*°(solute, H_2_O → D_2_O, 298 K)/kJ mol^−1^ = −1.09 (methane), −1.35 (ethane), −1.57 (propane)) and entropically unfavorable (−*T*Δ_t_*S*°/kJ mol^−1^ = 0.95 (methane), 1.23 (ethane), 1.47 (propane)), leading to very small Δ_t_*G*° (−0.10 up to −0.14 kJ mol^−1^ for these alkanes) [70,71]. Concurringly, the transfer enthalpies of tetraalkylammonium cations become more negative with increasing alkyl chain length [72,73]. One would therefore expect that the isotope substitution affects the thermodynamics of hydrophobically driven association considerably.

G. C. Kresheck determined the micellization enthalpy of alkyldimethylphosphine oxides in H_2_O and D_2_O over 288–352 K, together with the corresponding Δ_r_*C_p_*° values [74]. Micellization was found to be more endothermic by up to 1.3 kJ mol^−1^ in D_2_O, where slightly more negative heat capacity changes were also observed. Consistently, a subsequent study by Angarten and Loh on the micellization of nonyl monoglucoside reported marginally more positive enthalpies and entropies in D_2_O [75].

In a seminal study of solvent isotope effects on the cyclodextrin host reactions, Rekharsky and Inoue investigated the complexation of more than 30 neutral and charged guests with native α-cyclodextrin (**α-CD**) and native and modified β-cyclodextrin (**β-CD**) in H_2_O and D_2_O by calorimetry at 298 K [66]. Isotope substitution affected both Δ_r_*H*° and –*T*Δ_r_*S*°, with differences in complexation enthalpies reaching up to 1.9 kJ mol^−1^; however, these effects were largely compensated by the corresponding entropy changes. To the best of our knowledge, only the binding of camphor to **α-CD** has been examined at different temperatures in D_2_O and H_2_O using ITC, albeit in quite a narrow range (293−313 K) [76]. The reported results show the same compensation pattern: at 298 K, complexation was less exothermic in D_2_O, resulting in ΔΔ_r_*H*° = 2.4 and 5.2 kJ mol^−1^ for (+)- and (−)-camphor, while the −*T*ΔΔ_r_*S*° amounted to −4.8 and −7.2 kJ mol^−1^, leading to slightly more stable complexes in D_2_O. The reaction heat capacities were more negative in D_2_O by up to 49% for the (+)-enantiomer, while for (−)-camphor this amounted to only 15%.

Because cucurbiturils contain a larger number of less strongly associated water molecules than their cyclodextrin counterparts [77,78,79], a more pronounced isotope effect on binding thermodynamics might be expected for these particularly efficient receptors of hydrophobic and amphiphilic guests, such as α,ω-alkyldications [80]. However, ITC data concerning cucurbituril hosting properties in heavy water remain scarce. To date, only two studies have been reported, both at 298 K. Biedermann et al. found that the inclusion of indole, 2-naphthol, or 2,7-dihydroxynaphthalene derivatives into cucurbit[8]uril:methyl viologen (**CB8**:MV) complex was more exothermic in D_2_O (Δ_r_*H*°/kJ mol^−1^ = −58.8 (D_2_O) and −54.3 (H_2_O) for indole) [67]. The other study examined the binding of a tertiary amine and a Diels–Alder cycloadduct by cucurbit[7]uril (**CB7**); however, the complexation thermodynamic parameters were determined solely in D_2_O [81].

The pronounced variation of Δ_r_*H*° and Δ_r_*S*° with temperature in H_2_O is a thermodynamic fingerprint of cyclodextrin and cucurbituril inclusion reactions [40,82,83,84,85] and has been known to correlate with the extent of hydrophobic surface dehydration [86,87,88,89,90,91]. Considering this fact and the notable temperature effect on the organization of D_2_O and H_2_O [17,51,52,92], comparative calorimetric studies of the cyclodextrin and particularly cucurbituril complexation reactions involving larger guests whose solvation spheres encompass twenty or more water molecules (e.g., adamantane [93]) could be particularly informative.

**Figure 1:**
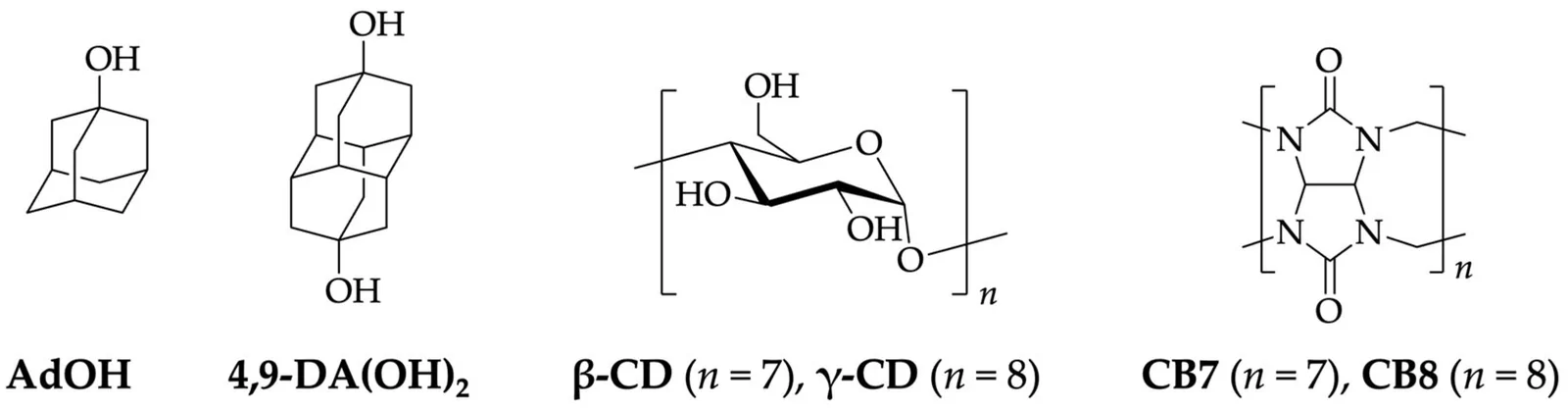
Structures of the investigated compounds.

Motivated by this literature gap, we decided to study the binding of adamantan-1-ol (**AdOH**) and diamantan-4,9-diol (**4,9-DA(OH)_2_**) to β- and γ-cyclodextrin (**β-CD** and **γ-CD**) as well as to their cucurbituril analogs (Figure 1) using isothermal titration microcalorimetry. Measurements with cucurbit[*n*]urils (*n* = 7 and 8, **CB7** and **CB8**) were performed over 278–338 K in H_2_O and D_2_O, whereas the binding studies in D_2_O complement the corresponding H_2_O data recently reported by our group [82,85].

## 2. Results and Discussion

### 2.1. Solvent Isotope Effect on Complex Stability

Representative microcalorimetric titrations of **4,9-DA(OH)_2_** with **β-CD** in D_2_O are presented in Figure 2, while the remaining ITC results are provided in Figures S1−S23. The normalized successive enthalpy changes were satisfactorily described by a 1:1 binding model (more details on the fitting procedure are given in Section 3.2), yielding the thermodynamic parameters listed in Table 1. For complexes with stability constants beyond the measurable range of direct ITC titration, only Δ_r_*H*° could be obtained.

In general, solvent isotope substitution has only a minor effect on the stability of complexes with cyclodextrins throughout the explored temperature range. At 298 K, Δlog *K*° (defined as log *K*°(D_2_O) − log *K*°(H_2_O)) amounts to +0.08 for **AdOH:β-CD** and +0.10 for **4,9-DA(OH)_2_:β-CD** (Table 1), consistent with previously reported stability increase in D_2_O [66]. In the case of **γ-CD** complexes, these differences are slightly larger (Δlog *K*° ranging from −0.14 to 0.21) and are the most pronounced for **4,9-DA(OH)_2_**, although, due to very low reaction heats and the resulting larger uncertainties, the variations cannot be unambiguously attributed to solvent substitution.

Given the fact that cucurbiturils, compared to their cyclodextrin analogs, contain more frustrated water molecules, which are also considerably less associated [79], one might expect a larger effect of H_2_O/D_2_O substitution on the reaction equilibrium constants for **CB**s than for **CD**s. However, the observed effects are quite similar. For the **AdOH:CB8** complex, Δlog *K*° at 298 K reaches −0.25, corresponding to *K*°(D_2_O)/*K*°(H_2_O) = 0.56. This value is close to that reported for methyl viologen binding to **CB8** (*K*°(D_2_O)/*K*°(H_2_O) = 0.50), which, to our knowledge, is the only other study comparing **CB8** binding affinities in H_2_O and D_2_O [67]. The effect of H_2_O/D_2_O substitution on the stability of **4,9-DA(OH)_2_**:**CB7** is even smaller. As shown in Table 1, the difference in log *K*° between H_2_O and D_2_O is comparable to the corresponding standard error of the mean.

### 2.2. Solvent Isotope Effect on the Reaction Enthalpy and Entropy

Prior to discussing the solvent isotope effect, it is worth revisiting the origins of enthalpic and entropic contributions to Δ_r_*G*° in the studied systems, especially since the seven chosen guest:host pairs span the range from strongly exothermic (enthalpically driven) to endothermic (entropically driven) binding (Table 1). The enthalpy of hydrophobic guest transfer from water to the macrocycle’s nonpolar cavity should be close to zero. Namely, the enthalpy of cyclohexane dehydration (33.2 kJ mol^−1^) and its condensation (−33.05 kJ mol^−1^), which can, in a first approximation, be taken to correspond to the enthalpy of its interaction with the cavity atoms of the host, nearly cancel each other out at 298 K [94,95]. On the other hand, the enthalpically beneficial expulsion of cavity water depends on the guest-host fit (extent of dehydration) and the degree of cavity water association [67,77,78,79,82,83]. Molecular dynamics simulations place the average number of hydrogen bonds per confined water molecule at 1.9 for **β-CD** against 2.2 for **γ-CD**, with 3.6 in the bulk [79], so its release is enthalpically more favorable for the smaller macrocycle, while cucurbituril cavities of comparable size contain more “frustrated” water molecules than **CD**s. Consequently, the inclusion of classically hydrated guests in cucurbiturils is strongly exothermic, less so in **β-CD**, and often endothermic in **γ-CD**.

A negative Δ_r_*S*° is often attributed to the formation of one species from two reactants, but can also stem from the entropic cost of hydrogen-bond formation upon cavity water expulsion [83], while an entropically positive contribution arises from the dehydration of classically hydrated guests.

As shown, the pronounced decrease in Δ_r_*H*° and Δ_r_*S*° with temperature is predominantly due to the melting of guest hydration shells and is therefore correlated with the extent of guest dehydration [82,83]. These changes can even shift the inclusion driving force for cyclodextrins [83,84,85,96]. Conversely, their complexation by **CB7** and **CB8** usually remains predominantly enthalpy-driven across the 278−338 K temperature range [83,84,96].

**Figure 3:**
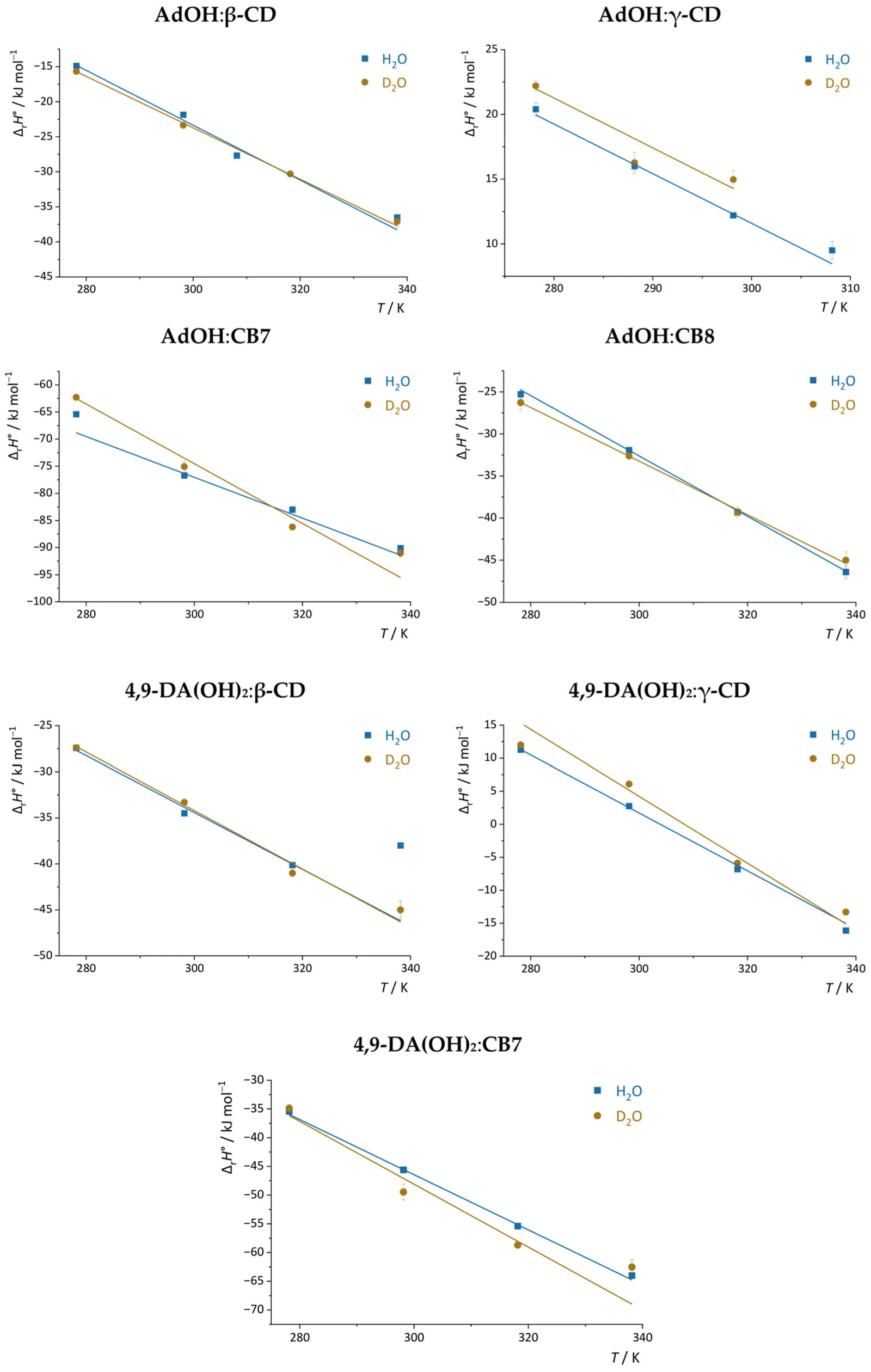
Temperature dependence of Δ_r_*H*°(guest:host) in H_2_O (blue squares) and D_2_O (golden circles). The lines correspond to the weighted linear regression fit of Δ_r_*H*°(*T*) for both systems.

The complexation enthalpies and entropies in some cases reveal a stronger effect of H_2_O/D_2_O substitution. Although the differences are rather small on the scale of the absolute values of reaction parameters for most complexes, they are largely outside the experimental uncertainty range. Taken across all seven complexes, the ΔΔ_r_*H*° (defined as Δ_r_*H*°(in D_2_O) − Δ_r_*H*°(in H_2_O)) ranges from −8.0 to 3.3 kJ mol^−1^ and depends on the host–guest pair, and the temperature (Figure 3). With the exception of one outlier (**4,9-DA(OH)_2_**:**β-CD** complex at 338 K), both ΔΔ_r_*H*° and −*T*ΔΔ_r_*S*° remain within ±5 kJ mol^−1^.

The data listed in Table 1 suggest that the effect of solvent isotope composition is somewhat more pronounced for cyclodextrins, especially in the case of **β-CD**, which also forms more stable complexes with both alcohols than the larger homolog. One can also observe that the differences in binding enthalpies and entropies between D_2_O and H_2_O for both **β-CD** and **γ-CD** increase as the temperature rises.

Considering that cucurbiturils contain more water molecules than their cyclodextrin analogues, and that these water molecules are also less strongly associated than those confined within the cavities of **CD**s, we expected a more pronounced medium effect on the binding thermodynamics. However, as demonstrated above, the differences in Δ_r_*H*° and –*T*Δ_r_*S*° in D_2_O and H_2_O were either comparable or even lower than those observed for cyclodextrin complexes. Nevertheless, several interesting trends for the seven- and eight-membered glycoluril-based macrocyclic hosts can be noticed. For **CB8**, the enthalpies of **AdOH** complexation in H_2_O and D_2_O are nearly identical above 278 K. In contrast, the Δ_r_*H*° values for **4,9-DA(OH)_2_** and **AdOH** complexation with **CB7** exhibit more complex temperature dependences. For **AdOH**, binding in H_2_O is more exothermic at lower temperatures, whereas the opposite holds at 318 and 338 K. A similar behavior is observed for **4,9-DA(OH)_2_** at 278 and 338 K, while at intermediate temperatures (298 and 318 K), ΔΔ_r_*H*° is negative.

The near constancy, or complete invariability, of the complex stability constants and consequently of Δ_r_*G*° upon transfer to D_2_O can be attributed to enthalpy–entropy compensation. However, we once again emphasize that the differences in Δ_r_*H*° and –*T*Δ_r_*S*° between D_2_O and H_2_O are generally rather small. This is particularly important because the complex stability constant and binding enthalpy are determined from nonlinear regression analysis of the Δ*H*°(*c*_G_, *c*_H_) dependence obtained in a single experiment and are therefore inherently correlated. Consequently, the fitted values and their associated uncertainties are not independent. Differences between the Δ_r_*H*° and –*T*Δ_r_*S*° values obtained in D_2_O and H_2_O should therefore be interpreted with caution, particularly when they are comparable to the uncertainties of the fitted parameters.

To gain a better insight into the solvent effect on the thermodynamic complexation parameters listed in Table 1, error propagation analysis was conducted according to Equations (S1)–(S5) in the Supplementary Materials. The differences between the mean values and estimates of the standard errors of these differences (s(Δlog *K*°), s(ΔΔ_r_*X*°)) are given in Table 2. Out of 26 ΔΔ_r_*H*° values, 19 exceed their combined standard error, with 15 of them exceeding 2*s*(ΔΔ_r_*H*°). The reaction Gibbs energies behave differently: only nine of the 21 ΔΔ_r_*G*° values exceed 2*s*(ΔΔ_r_*G*°), whereas the remaining differences are statistically non-significant according to this criterion. The seven reaction enthalpy differences that do not exceed their combined uncertainties are all (in absolute value) below 1.0 kJ mol^−1^. Four of these correspond to **CB7** and **CB8** complexes, for which the combined standard errors are the largest, reaching 1.3 kJ mol^−1^ for **AdOH**:**CB8** at 338 K. The differences in –*T*Δ_r_*S*° exceed twice their standard errors for 13 out of the 21 values, similarly to the differences in reaction enthalpy, reflecting the observed enthalpy–entropy compensation. The s(ΔΔ_r_*S*°) values are again the largest for the formation of the **CB7** and **CB8** complexes, indicating, together with the corresponding results for ΔΔ_r_*H*°, that the non-significant differences in Δ_r_*X*° for the inclusion within cucurbiturils could be partly due to experimental error.

The differences between the thermodynamic parameters determined for the reactions in H_2_O and D_2_O were additionally assessed using two-sided Welch’s *t*-test. The results are presented in Table S1 and are consistent with the considerations discussed above. The differences between the mean stability constants are not statistically significant at the *α* = 0.05 significance level for most of the systems, with the exception of **4,9-DA(OH)_2_**:**γ-CD**. However, the reaction enthalpies and entropies differ significantly in a considerable number of cases, again suggesting the above-mentioned enthalpy–entropy compensation in some instances.

The standard isobaric reaction heat capacities in both solvents, obtained by processing the Δ_r_*H*°(*T*) data using a weighted linear regression procedure, with Δ_r_*C_p_*° corresponding to the slope of the obtained dependencies (Figures S24–S30), are also listed in Table 1. The obtained values are in all cases strongly negative (−307 to −545 J K^−1^ mol^−1^) and, except for **AdOH**:**CB8** and **CB7** complexes, very similar in both H_2_O and D_2_O. However, as seen in Table 1, based on the linearization of Δ_r_*H*°(*T*), the hosting of **AdOH** and **4,9-DA(OH)_2_** with cucurbit[7]uril resulted in larger Δ_r_*C_p_*° uncertainties in comparison to most of the other examined host–guest pairs, so the observed differences in heat capacities for the binding with this particular host in D_2_O and H_2_O must be interpreted carefully. Note that our results are quite different from those for the binding of camphor by **α-CD**, where differences of ΔΔ_r_*C_p_*° = −306 to −100 J K^−1^ mol^−1^ were obtained for the 1:2 (guest:host) complexes of (+)- and (−)-camphor with **α-CD**, respectively [76].

The most negative Δ_r_*C_p_*° values were obtained for the binding of **4,9-DA(OH)_2_** with **CB7**, followed by the reaction of this diamantane-based guest with **γ-CD**. This finding is in accord with the previously observed correlation of reaction heat capacity and the size of buried classically hydrated non-polar guest surface [86,87,88,90]. Both **γ-CD,** in which **4,9-DA(OH)_2_** can assume a somewhat tilted conformation, and the barrel like-elongated cavity of **CB7** lead to more extensive dehydration of the guest backbone compared to its inclusion inside **β-CD** [82]. It is worth noting that Grimm et al. [84] obtained very similar Δ_r_*C_p_*° values for the complexation of **AdOH** and **4,9-DA(OH)_2_** with **β-CD**, **CB7** and **CB8** in H_2_O, which supports the reliability of the results presented herein.

## 3. Materials and Methods

### 3.1. Materials

Adamantan-1-ol (**AdOH**; Sigma-Aldrich, Steinheim, Germany, 99%) and cucurbit[7]uril (**CB7**; Sigma-Aldrich, Steinheim, Germany, hydrate) were used as received. β-Cyclodextrin (**β-CD**; Sigma-Aldrich, Steinheim, Germany, HPLC grade, ≥98%) and γ-cyclodextrin (**γ-CD**; TCI Chemicals, Tokyo, Japan, >99.9%) were dried at 150 °C prior to use. Diamantan-4,9-diol (**4,9-DA(OH)_2_**) was synthesized and purified according to the previously published procedure [97], as was cucurbit[8]uril (**CB8**) [98]. 18-Crown-6 ether (**18C6**; Sigma-Aldrich, Steinheim, Germany, 99%) and BaCl_2_ (Sigma-Aldrich, Steinheim, Germany, 99.9%) were used for calorimeter calibration. Solutions were prepared by direct weighing of the solids and dissolution in D_2_O (Sigma-Aldrich, Steinheim, Germany, >99.9% D) or Milli-Q water (Merck Millipore, Burlington, MA, USA).

### 3.2. Isothermal Titration Microcalorimetry

Calorimetric measurements were carried out on a MicroCal PEAQ-ITC microcalorimeter (Malvern Panalytical, Malvern, UK, *V*_cell_ = 0.205 mL) at temperatures ranging from 278 to 338 K. The host solutions (*c* = 1.01 × 10^−3^ to 1.10 × 10^−2^ mol dm^−3^) were added stepwise to the guest solution (*c*_0_ = 6.01 × 10^−5^ to 1.55 × 10^−4^ mol dm^−3^), with the exception of sparingly soluble **CB8** (*c*_0_ = 3.21 × 10^−5^ mol dm^−3^) which was titrated with **AdOH** (*c* = 3.08 × 10^−4^ mol dm^−3^). The aqueous solubility of **CB8** is reported to be below 0.01 mmol dm^−3^ (ref. [99,100]); to the best of our knowledge, no corresponding value has been reported for D_2_O. Blank experiments were carried out, and the heats of titrant dilution were subtracted. Nonlinear regression analysis of the thermograms was carried out with the 1:1 stoichiometric model by using PEAQ-ITC Analysis Software (version 1.30, Malvern Panalytical, Malvern, UK) supplied by the manufacturer. The complex stoichiometry was fixed at *N* = 1 (one-set-of-sites model). Further, the sigmoidal shape of the titration curves in the case of tighter binding (log *K*° above 4), with the inflection point at a host-to-guest molar ratio of approximately 1, clearly indicated 1:1 stoichiometry of the formed complexes. Fitting the data with a sequential two-site model, which accounts for a higher-stoichiometry complex, did not improve agreement between fitted and experimental values; therefore, the simplest 1:1 model was retained. By fixing the stoichiometry at 1 and reaching saturation, complex stability constants could reliably be determined regardless of a low *c* parameter [101]. For complexes of very high stability (log *K*° ≥ 7.5) only Δ_r_*H*° was determined. All titrations were performed at least in triplicate. Standard isobaric reaction heat capacities were obtained by weighted linear regression of Δ_r_*H*°(*T*) using Origin 7.0 (OriginLab Corporation, Northampton, MA, USA).

Comparison of the mean values of the thermodynamic parameters determined in H_2_O and D_2_O was performed by means of Welch’s *t*-test using Origin 7.0 (OriginLab Corporation, Northampton, MA, USA).

To assess the impact of OH-OD exchange on the determined thermodynamic reaction parameters, isotope exchange was monitored by ^1^H NMR (Section 3.3) and the titrations were repeated with the five-day-old solutions. The differences in parameters were below the corresponding experimental uncertainties, suggesting that the H/D exchange of the solute OH group protons affects the thermodynamic reaction parameters very weakly or not at all.

The reliability of the calorimeter was checked by means of the complexation of Ba^2+^ with **18C6** at 298 K as recommended by Briggner and Wadsö (*K* = 5.9 × 10^3^ dm^3^ mol^−1^, Δ_r_*G*° = −21.5 kJ mol^−1^, Δ_r_*H*° = −31.4 kJ mol^−1^, Δ_r_*S*° = −33.1 J K^−1^ mol^−1^) [102,103]. The values obtained were in very good agreement with the literature values: *K* = (4.9 ± 0.3) × 10^3^ dm^3^ mol^−1^, Δ_r_*G*° = (−21.1 ± 0.1) kJ mol^−1^, Δ_r_*H*° = (−30.1 ± 0.1) kJ mol^−1^, and Δ_r_*S*° = (−30.2 ± 0.9) J K^−1^ mol^−1^.

### 3.3. ^1^H NMR Spectroscopy

^1^H NMR experiments were performed in D_2_O at 298 K on a Bruker Avance III HD 400 MHz/54 mm spectrometer (Bruker BioSpin GmbH, Rheinstetten, Germany) equipped with an inverse broadband room-temperature probe (5 mm PA BBI 1H/D–BB). Spectra were recorded with standard Bruker pulse sequences and processed with TopSpin software (version 3.6.2, Bruker BioSpin, Rheinstetten, Germany) supplied by the manufacturer. To test whether the OH group proton–deuterium exchange affects the thermodynamic reaction parameters, ^1^H NMR spectra of the representative solutions were recorded upon their preparation and daily for five days.

## 4. Conclusions

Complexation of two aliphatic alcohols, adamantan-1-ol and diamantan-4,9-diol, by four macrocyclic receptors (β- and γ-cyclodextrin, cucurbit[7]uril and cucurbit[8]uril) was studied calorimetrically in H_2_O and D_2_O over a wide temperature range (278−338 K). By comparing the differences between the mean values of log *K*°(H_2_O) and log *K*°(D_2_O) with the standard errors of these differences, as well as by assessing their statistical significance using the corresponding *t*-tests, we found that solvent deuteration has almost no effect on complex stability for most of the investigated systems. The change in log *K*°(298 K) is below 0.1 for **β-CD** complexes, and the largest effect was seen for the complex of **AdOH** with **CB8** (*K*°(D_2_O)/*K*°(H_2_O) = 0.56). Contrary to the expectation that the solvent (H/D) isotope effect on the thermodynamic complexation parameters would be larger for cucurbiturils, which contain more “enthalpically frustrated” water than cyclodextrins, this was not the case. In fact, the effect is slightly more pronounced among cyclodextrins, particularly for the formation of the **4,9-DA(OH)_2_**:**γ-CD** complex.

For many explored guest:host pairs, the ΔΔ_r_*H*° and −*T*ΔΔ_r_*S*° lie outside their experimental uncertainties, yet they remain within ±5 kJ mol^−1^ (with the exception of one outlier, the **4,9-DA(OH)_2_**:**β-CD** complex at 338 K). Due to the partial enthalpy–entropy compensation, the ΔΔ_r_*G*° between D_2_O and H_2_O does not exceed 1.4 kJ mol^−1^ across all systems. These differences are largest at the ends of the examined temperature interval, indicating the importance of conducting such studies across several temperatures. The reaction heat capacities are negative in both solvents (−307 to −545 J K^−1^ mol^−1^) and differ within the uncertainty for five out of seven guest:host pairs.

In practical terms, we have found that for hydrophobically driven complexation involving cyclodextrins and cucurbiturils, the complex stability determined in D_2_O (as is most often the case in ^1^H NMR experiments) can be transferred to H_2_O with low uncertainty. However, the same does not necessarily hold for Δ_r_*H*° and −*T*Δ_r_*S*° which varied by up to 9 kJ mol^−1^ for the **4,9-DA(OH)_2_** complex with **β-CD** at 338 K.

Finally, the findings presented herein may be of practical relevance to medicinal and pharmaceutical applications, given the importance of the stabilization and solubilization properties of the examined hosts. This holds true particularly for cyclodextrins and their derivatives [46,47,48,49,82,96,104], whose excipient properties are often evaluated by routine NMR experiments in D_2_O at 298 K, while what is actually most relevant is their hosting ability in H_2_O at formulation storage temperature (customarily 278 K) and body temperature (310 K).

Complexation by these hosts also increases the apparent aqueous solubility of poorly soluble guests, an effect documented both for cyclodextrins [47,48,49] and for cucurbiturils [100,105,106,107,108,109], for which encapsulation increased the solubility of sparingly soluble drugs by one to three orders of magnitude. This should also hold for the guests investigated herein, whereby the differences in the complex stability constants determined in H_2_O and D_2_O indicate a negligible solvent isotope effect on the extent of solubilization.

## Figures and Tables

**Figure 2:**
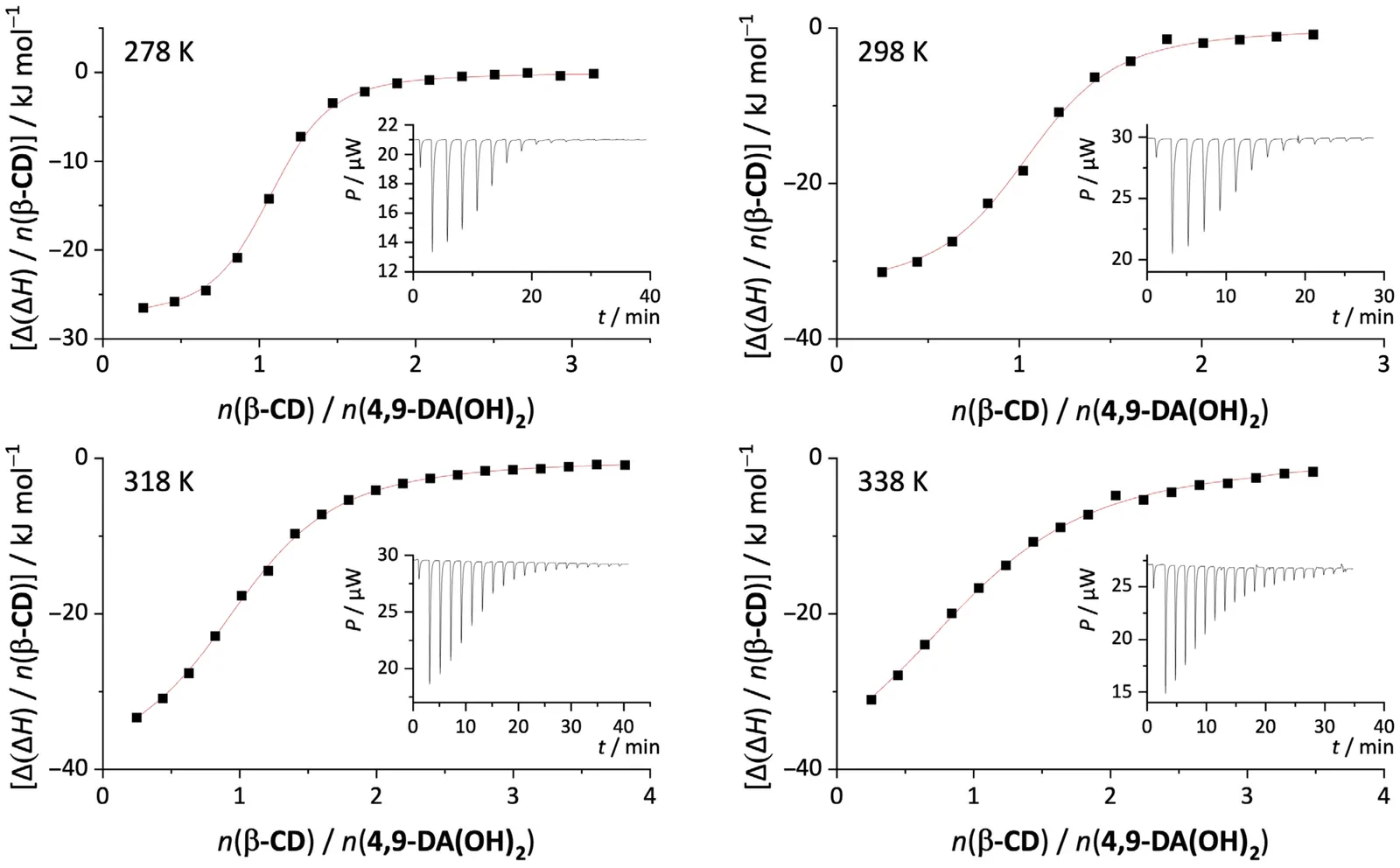
Microcalorimetric titrations of **4,9-DA(OH)_2_** (*c*_0_ = 1.25 × 10^−4^ mol dm^−3^) with **β-CD** (*c* = 4.94 × 10^−3^ mol dm^−3^) in D_2_O at *T* = 278–338 K. Dependences of the normalized successive enthalpy changes on the host-to-guest molar ratio are given as main figures, and the corresponding titration thermograms as inserts. ■ experimental; − calculated.

**Table 1:** Standard thermodynamic parameters for complexation of **AdOH** and **4,9-DA(OH)_2_** with cyclodextrins and cucurbiturils in D_2_O and H_2_O at *T* = 278−338 K ^a^.

Guest	Host	*T*/K	log *K*°		Δ_r_*G*°/kJ mol^−1^		Δ_r_*H*°/kJ mol^−1^		−*T*Δ_r_*S*°/kJ mol^−1^		Δ_r_*C_p_*°/J K^−1^ mol^−1^	
			D_2_O	H_2_O ^b^	D_2_O	H_2_O ^b^	D_2_O	H_2_O ^b^	D_2_O	H_2_O ^b^	D_2_O	H_2_O ^b^
**AdOH**	**β-CD**	278	4.93(2)	4.90(1)	−26.3(1)	−26.08(1)	−15.7(1)	−14.88(5)	−10.6(2)	−11.23(5)	−366(7)	−391(36) ^c^
		298	4.70(2)	4.62(1)	−26.8(1)	−26.38(3)	−23.4(3)	−21.86(6)	−3.5(4)	−4.53(5)		
		308		4.49(1)		−26.46(3)		−27.68(7)		1.20(9)		
		318	4.46(2)		−27.2(1)		−30.3(3)		3.1(4)			
		338	4.12(1)	4.06(1)	−26.7(1)	−26.26(4)	−37.1(8)	−36.5(2)	10.4(9)	10.2(3)		
	**γ-CD**	278	2.55(4)	2.39(2)	−13.6(2)	−12.7(1)	22.2(4)	20.4(5)	−35.8(4)	−33.1(4)	−384(100)	−380(27)
		288	2.58(5)	2.54(1)	−14.3(2)	−14.00(3)	16.3(8)	16.0(3)	−30.6(6)	−30.0(4)		
		298	2.65(2)	2.62(1)	−15.1(1)	−14.9(1)	15.0(7)	12.2(2)	−30.1(6)	−27.1(1)		
	**CB7**	278	− ^d^	≳7.5	− ^d^	− ^d^	−62.3(2)	−65.4(4)	− ^d^	− ^d^	−545(49)	−370(62)
		298	− ^d^	≳7.5	− ^d^	− ^d^	−75.1(9)	−76.70(1)	− ^d^	− ^d^		
		318	− ^d^	≳7.5	− ^d^	− ^d^	−86.2(4)	−83.0(4)	− ^d^	− ^d^		
		338	− ^d^	≳7.5	− ^d^	− ^d^	−91.0(7)	−90.1(4)	− ^d^	− ^d^		
	**CB8**	278	− ^d^	7.4(1)	− ^d^	−39.4(8)	−26.3(9)	−25.3(6)	− ^d^	−14(1)	−333(3)	−359(10)
		298	7.09(6)	7.34(5)	−40.5(3)	−41.9(3)	−32.6(2)	−31.93(3)	−7.9(2)	−10.0(3)		
		318	7.1(2)	7.02(6)	−43(2)	−42.7(4)	−39.3(6)	−39.3(4)	−4(2)	−3.5(7)		
		338	7.0(1)	6.84(4)	−45.1(6)	−44.3(2)	−45(1)	−46.4(8)	0(2)	2(1)		
**4,9-DA(OH)_2_**	**β-CD**	278	5.45(2)	5.41(1)	−29.03(9)	−28.79(1)	−27.4(2)	−27.41(7)	−1.6(2)	−1.5(1)	−317(20)	−307(39)
		298	5.12(2)	5.02(1)	−29.23(9)	−28.67(4)	−33.3(2)	−34.5(2)	4.1(2)	5.8(2)		
		318	4.70(1)	4.67(1)	−28.63(7)	−28.45(6)	−41.0(4)	−40.13(3)	12.4(5)	11.68(8)		
		338	4.33(2)	4.51(1)	−28.0(1)	−29.17(5)	−46(1)	−38.0(3)	18(1)	8.9(3)		
	**γ-CD**	278	3.70(3)	3.54(1)	−19.7(2)	−18.83(1)	12.0(3)	11.27(3)	−31.7(2)	−30.13(3)	−507(64)	−440(11)
		298	3.5(1)	3.64(3)	−20.0(3)	−20.8(2)	6.1(1)	2.76(7)	−26.1(2)	−23.5(1)		
		318	3.66(1)	3.53(4)	−22.30(6)	−21.5(2)	−5.9(1)	−6.8(3)	−16.4(2)	−14.8(5)		
		338	3.68(1)	3.47(2)	−23.87(9)	−22.5(1)	−13.3(3)	−16.1(3)	−10.5(3)	−6.4(4)		
	**CB7**	278	7.00(1)	6.9(1)	−37.33(3)	−37.0(5)	−34.9(7)	−35.4(4)	−2.5(7)	−1.7(9)	−545(75)	−482(11)
		298	7.1(1)	7.2(1)	−40.7(7)	−41.0(6)	−50(1)	−45.6(1)	9(2)	4.5(8)		
		318	7.20(6)	7.09(9)	−43.9(4)	−43.2(5)	−58.7(4)	−55.4(3)	14.8(7)	12.2(6)		
		338	7.10(7)	6.99(2)	−46.0(5)	−45.2(1)	−63(1)	−64.0(5)	17(2)	19.0(5)		

^a^ Values refer to the process G + H ⇄ GH; the standard error of the mean (*N* = 3–5) is given in parentheses as the uncertainty of the last significant digit. ^b^ From ref. [82], with the exception of **AdOH**:**β-CD** (ref. [85]). ^c^ Obtained by weighted linear regression analysis of the data from ref. [85]. ^d^ Could not be determined by means of direct microcalorimetric titrations.

**Table 2:** Solvent isotope effect on the thermodynamic parameters of complexation of **AdOH** and **4,9-DA(OH)_2_** with cyclodextrins and cucurbiturils at *T* = 278−338 K, given as Δlog *K*° = log *K*°(D_2_O) − log *K*°(H_2_O) and ΔΔ_r_*X°* = Δ_r_*X°*(D_2_O) − Δ_r_*X°*(H_2_O), where *X* = *G*, *H*, and *S* ^a^.

Guest	Host	*T*/K	Δlog *K*°	ΔΔ_r_*G*°/kJ mol^−1^	ΔΔ_r_*H*°/kJ mol^−1^	−*T*ΔΔ_r_*S*°/kJ mol^−1^
**AdOH**	**β-CD**	278	0.03(2)	−0.2(1)	−0.8(1)	0.7(2)
		298	0.08(2)	−0.5(1)	−1.5(3)	1.1(4)
		338	0.06(1)	−0.39(9)	−0.6(8)	0.2(9)
	**γ-CD**	278	0.16(4)	−0.9(2)	1.8(6)	−2.7(6)
		288	0.04(5)	−0.2(3)	0.3(9)	−0.5(6)
		298	0.03(2)	−0.2(1)	2.8(7)	−3.0(6)
	**CB7**	278	−	−	3.1(4)	−
		298	−	−	1.6(9)	−
		318	−	−	−3.2(6)	−
		338	−	−	−0.9(8)	−
	**CB8**	278	−	−	−1(1)	−
		298	−0.25(8)	1.4(4)	−0.7(2)	2.1(4)
		318	0.1(2)	0(1)	0.0(7)	0(2)
		338	0.2(1)	−1.0(7)	1(1)	−2(2)
**4,9-DA(OH)_2_**	**β-CD**	278	0.04(2)	−0.2(1)	0.0(2)	−0.2(2)
		298	0.10(2)	−0.6(1)	1.2(3)	−1.8(3)
		318	0.03(1)	−0.18(9)	−0.9(4)	0.7(4)
		338	−0.18(2)	1.2(1)	−8(1)	9(1)
	**γ-CD**	278	0.16(3)	−0.9(2)	0.7(3)	−1.6(2)
		298	−0.1(1)	0.8(6)	3.3(1)	−2.5(5)
		318	0.13(4)	−0.8(3)	0.9(3)	−1.7(5)
		338	0.21(2)	−1.4(1)	2.8(4)	−4.2(5)
	**CB7**	278	0.1(1)	−0.5(5)	0.5(8)	−1(1)
		298	−0.1(1)	0.6(8)	−4(1)	5(2)
		318	0.1(1)	−0.7(7)	−3.3(5)	2.6(9)
		338	0.11(7)	−0.7(5)	1(1)	−2(1)

^a^ Calculated from the values listed in Table 1; the uncertainties were propagated according to Equations (S1)–(S5) in the Supplementary Materials and are given in parentheses as the uncertainty of the last significant digit.

## Data Availability

The data presented in this study are available in the article and Supplementary Materials.

## Supplementary Materials

The following supporting information can be downloaded at: https://www.mdpi.com/article/10.3390/molecules31193483/s1, Figures S1−S23: Microcalorimetric titrations of **AdOH** and **4,9-DA(OH)_2_** with **β-CD**, **γ-CD**, **CB7**, and **CB8** in D_2_O; Figures S24−S30: Temperature dependence of the standard thermodynamic reaction parameters for complexation of all guests with all hosts. Table S1: *p*-values of Welch’s *t*-test for the comparison of mean log *K*°, Δ_r_*H*°, and −*T*Δ_r_*S*° determined in H_2_O and D_2_O.

## Abbreviations

The following abbreviations are used in this manuscript:

**AdOH**	adamantan-1-ol
**4,9-DA(OH)_2_**	diamantan-4,9-diol
**α-CD**	α-cyclodextrin
**β-CD**	β-cyclodextrin
**γ-CD**	γ-cyclodextrin
**CB**	cucurbituril
**CB7**	cucurbit[7]uril
**CB8**	cucurbit[8]uril
**18C6**	18-crown-6 ether
ITC	isothermal titration calorimetry
NMR	nuclear magnetic resonance
CMC	critical micellization concentration
