# Supplementary material for: Solvent Isotope Effect on the Thermodynamics of Complexation with Cyclodextrins and Cucurbiturils: An ITC Study in H2O and D2O
Source: Molecules. 2026 Sep 29;31(19):3483. doi: 10.3390/molecules31193483 (PMC13666925; doi:10.3390/molecules31193483)
Supplement: Supplementary file 1 [file molecules-31-03483-s001.zip › molecules-4567070-supplementary.pdf]

Solvent Isotope Effect on the Thermodynamics of Complexation with Cyclodextrins and Cucurbiturils: An ITC Study in H_2O and D_2O

Josip Požar [1], Marija Alešković [2], Marina Šekutor [2], Vladislav Tomišić [1], Andrea Usenik [1,*]

[1] Department of Chemistry, University of Zagreb Faculty of Science, Horvatovac 102A, HR-10000 Zagreb, Croatia; pozar@chem.pmf.hr (J.P.), vtomisic@chem.pmf.hr (V.T.), ausenik.chem@pmf.hr (A.U.)
[2] Department of Organic Chemistry and Biochemistry, Ruđer Bošković Institute, Bijenička 54, HR-10000 Zagreb, Croatia; marija.aleskovic@irb.hr (M.A.), msekutor@irb.hr (M.Š.)
* Correspondence: ausenik.chem@pmf.hr; ORCID: 0000-0003-1995-8705

SUPPLEMENTARY MATERIALS

1. Experimental data

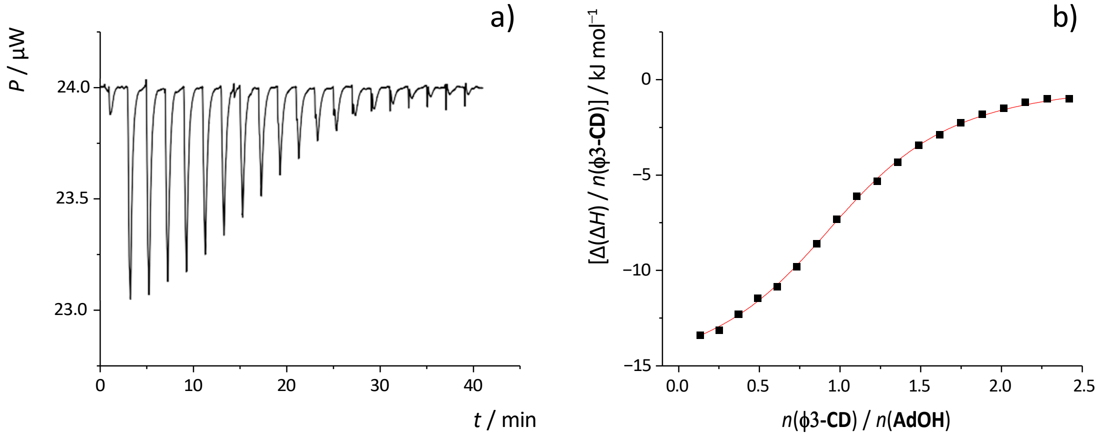

Figure S1. Microcalorimetric titration of **AdOH** (c_0 = 1.10 × 10^{-4} mol dm^{-3}) with **β-CD** (c = 9.46 × 10^{-3} mol dm^{-3}) in D_2O at 278 K. a) Thermogram. b) Dependence of the normalized successive enthalpy change on the host-to-guest molar ratio. ■ experimental; − calculated.

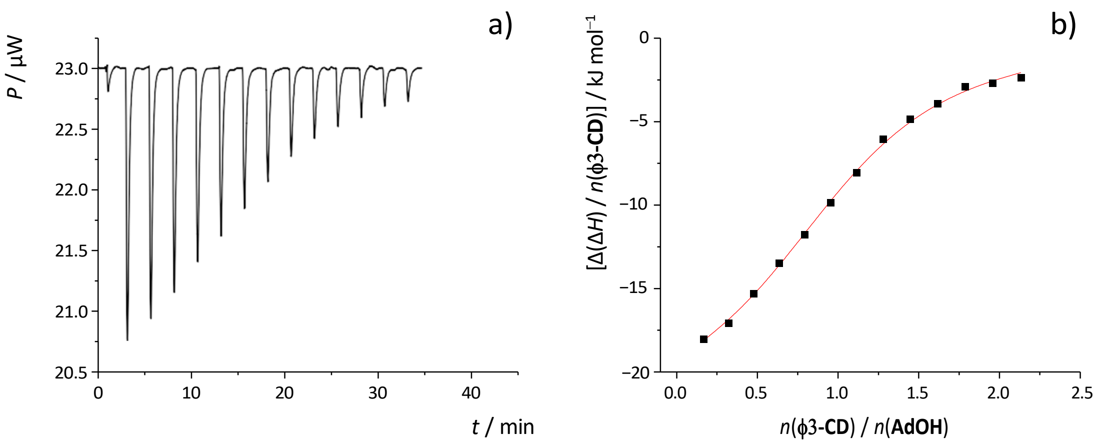

Figure S2. Microcalorimetric titration of **AdOH** (c_0 = 8.03 × 10^{-5} mol dm^{-3}) with **β-CD** (c = 9.46 × 10^{-3} mol dm^{-3}) in D_2O at 298 K. a) Thermogram. b) Dependence of the normalized successive enthalpy change on the host-to-guest molar ratio. ■ experimental; – calculated.

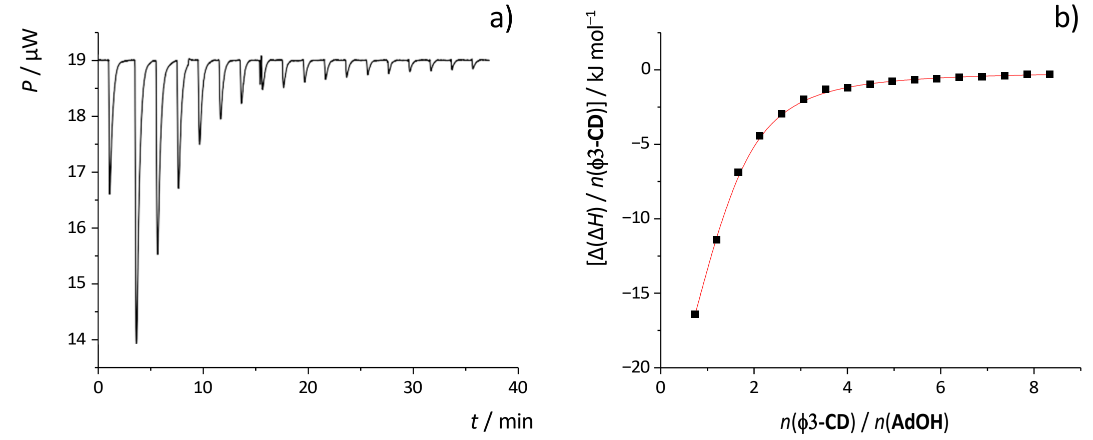

Figure S3. Microcalorimetric titration of **AdOH** (c_0 = 8.16 × 10^{-5} mol dm^{-3}) with **β-CD** (c = 1.00 × 10^{-2} mol dm^{-3}) in D_2O at 318 K. a) Thermogram. b) Dependence of the normalized successive enthalpy change on the host-to-guest molar ratio. ■ experimental; – calculated.

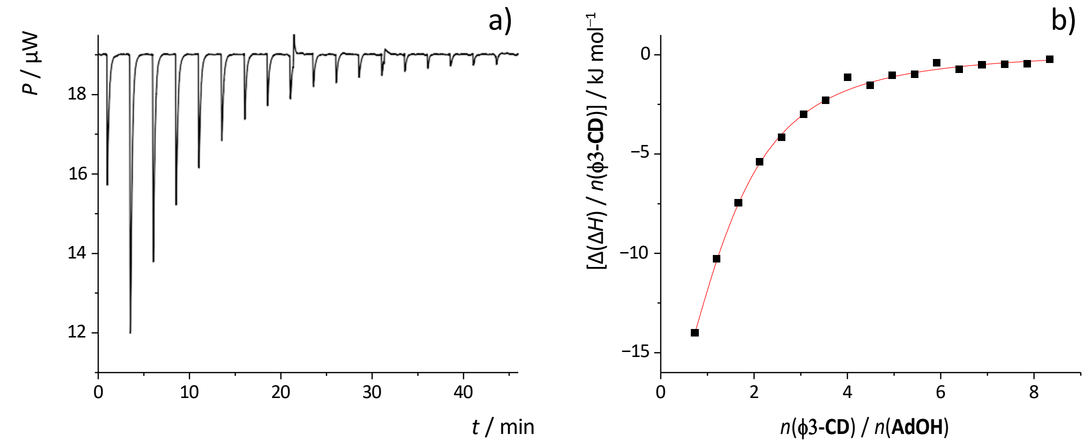

Figure S4. Microcalorimetric titration of **AdOH** (c_0 = 8.16 × 10^{-5} mol dm^{-3}) with **β-CD** (c = 1.00 × 10^{-2} mol dm^{-3}) in D_2O at 338 K. a) Thermogram. b) Dependence of the normalized successive enthalpy change on the host-to-guest molar ratio. ■ experimental; − calculated.

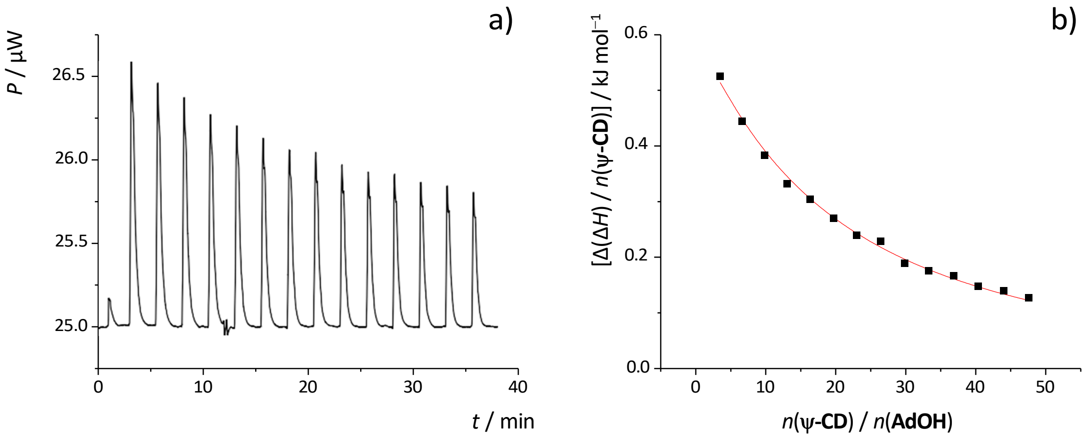

Figure S5. Microcalorimetric titration of **AdOH** (c_0 = 8.29 × 10^{-5} mol dm^{-3}) with **γ-CD** (c = 2.02 × 10^{-2} mol dm^{-3}) in D_2O at 278 K. a) Thermogram. b) Dependence of the normalized successive enthalpy change on the host-to-guest molar ratio. ■ experimental; − calculated.

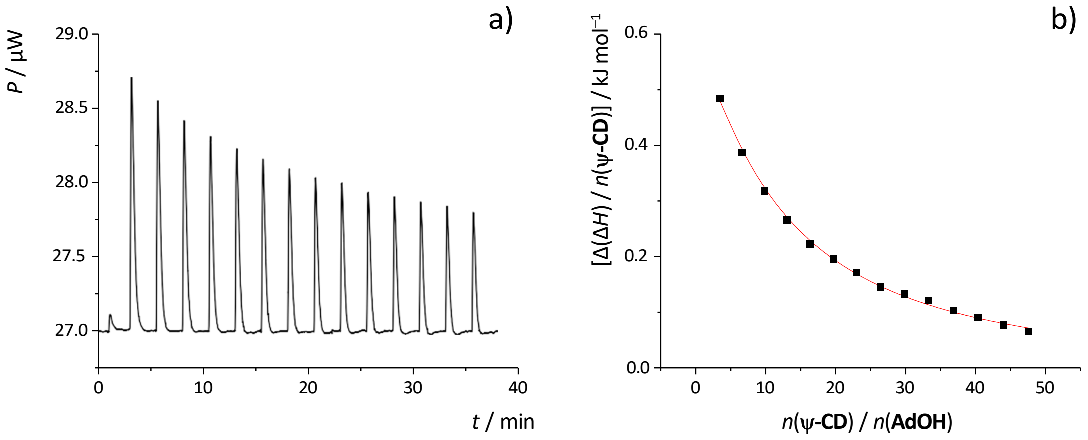

Figure S6. Microcalorimetric titration of **AdOH** (c_0 = 8.29 × 10^{-5} mol dm^{-3}) with **γ-CD** (c = 2.02 × 10^{-2} mol dm^{-3}) in D_2O at 288 K. a) Thermogram. b) Dependence of the normalized successive enthalpy change on the host-to-guest molar ratio. ■ experimental; – calculated.

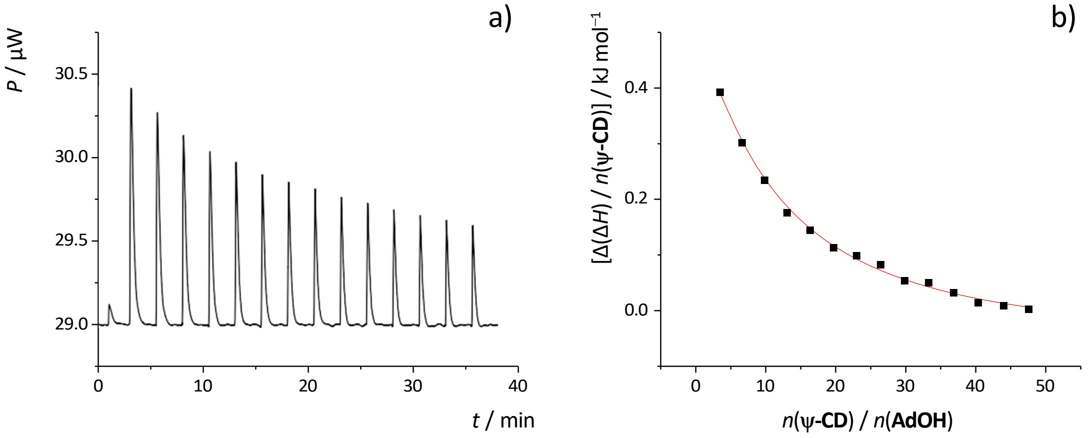

Figure S7. Microcalorimetric titration of **AdOH** (c_0 = 8.29 × 10^{-5} mol dm^{-3}) with **γ-CD** (c = 2.02 × 10^{-2} mol dm^{-3}) in D_2O at 298 K. a) Thermogram. b) Dependence of the normalized successive enthalpy change on the host-to-guest molar ratio. ■ experimental; – calculated.

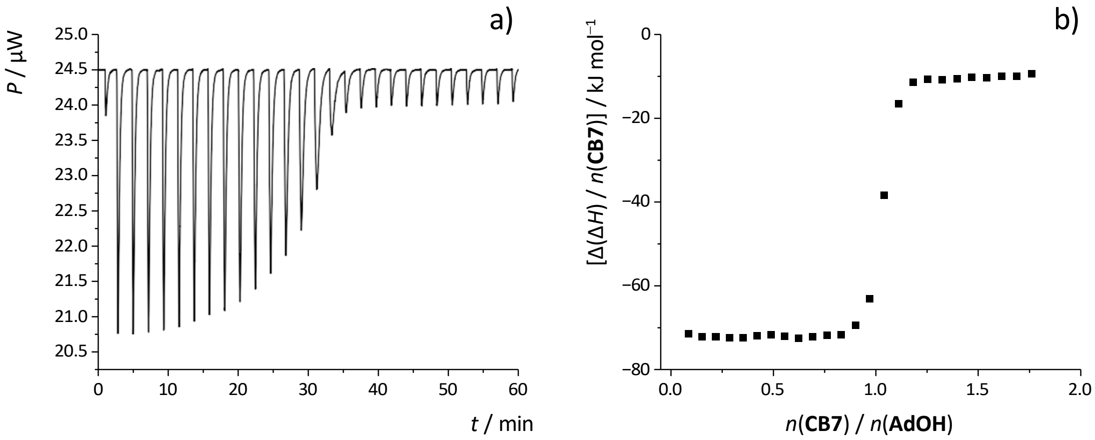

Figure S8. Microcalorimetric titration of **AdOH** (c_0 = 8.29 × 10−5 mol dm−3) with **CB7** (c = 1.15 × 10−3 mol dm−3) in D_2O at 278 K. a) Thermogram. b) Dependence of the normalized successive enthalpy change on the host-to-guest molar ratio.

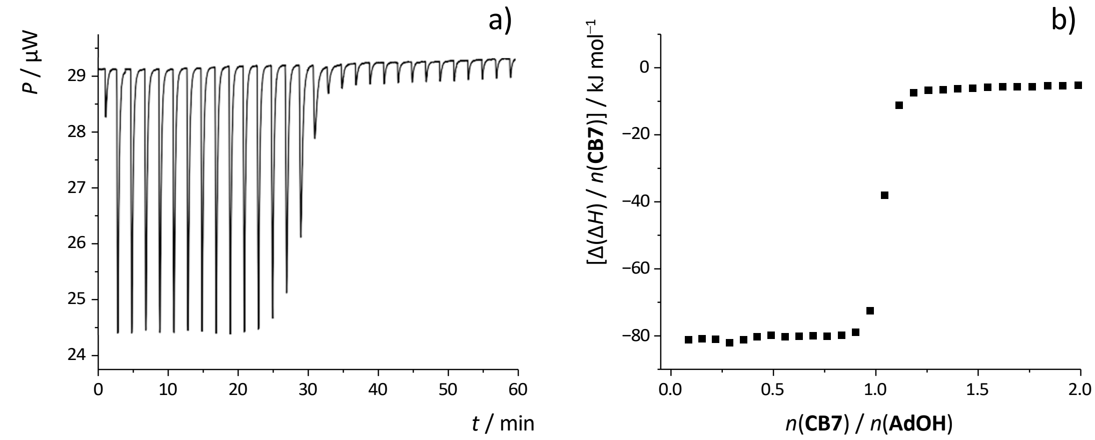

Figure S9. Microcalorimetric titration of **AdOH** (c_0 = 8.29 × 10−5 mol dm−3) with **CB7** (c = 1.15 × 10−3 mol dm−3) in D_2O at 298 K. a) Thermogram. b) Dependence of the normalized successive enthalpy change on the host-to-guest molar ratio.

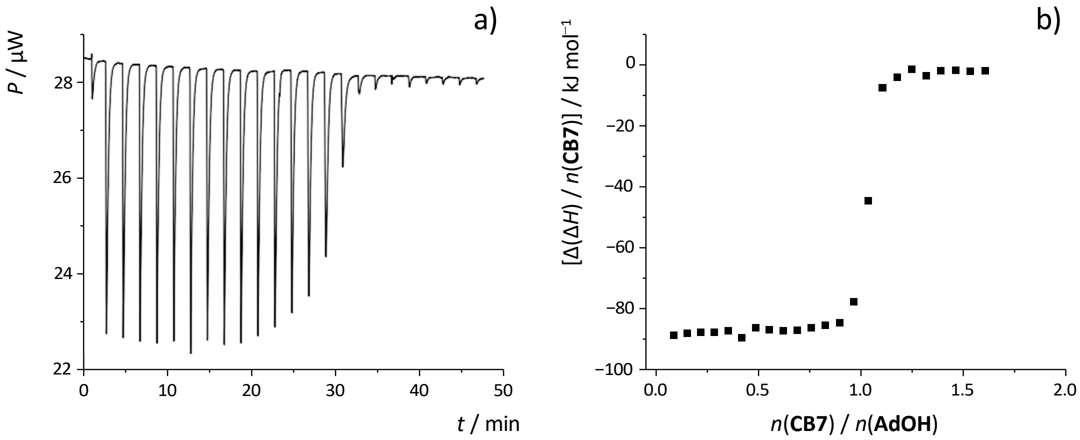

Figure S10. Microcalorimetric titration of **AdOH** (c_0 = 8.29 × 10^{-5} mol dm^{-3}) with **CB7** (c = 1.15 × 10^{-3} mol dm^{-3}) in D_2O at 318 K. a) Thermogram. b) Dependence of the normalized successive enthalpy change on the host-to-guest molar ratio.

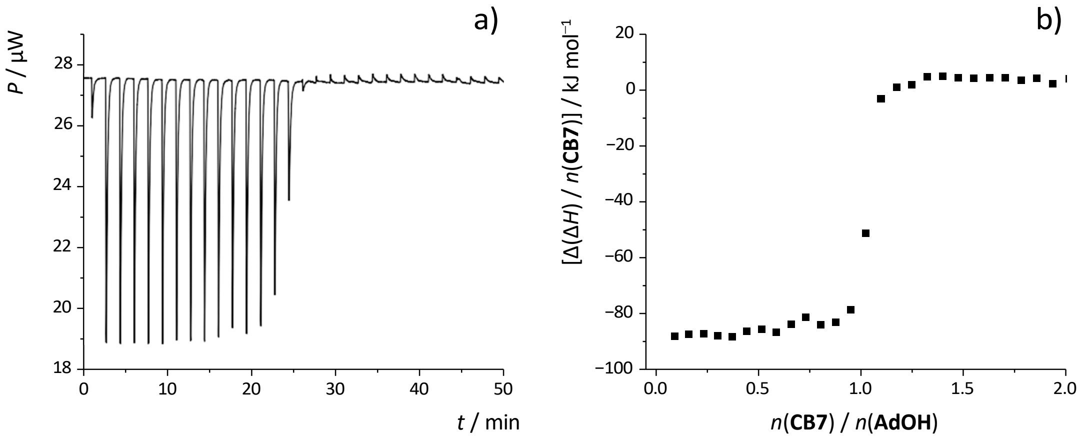

Figure S11. Microcalorimetric titration of **AdOH** (c_0 = 8.29 × 10^{-5} mol dm^{-3}) with **CB7** (c = 1.15 × 10^{-3} mol dm^{-3}) in D_2O at 338 K. a) Thermogram. b) Dependence of the normalized successive enthalpy change on the host-to-guest molar ratio.

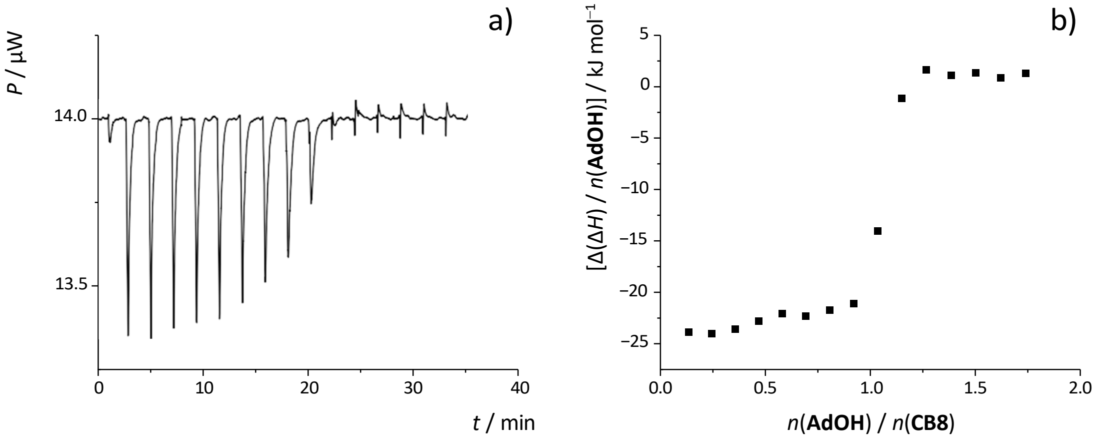

Figure S12. Microcalorimetric titration of **CB8** (c_0 = 3.21 × 10^{-5} mol dm^{-3}) with **AdOH** (c = 3.08 × 10^{-4} mol dm^{-3}) in D_2O at 278 K. a) Thermogram. b) Dependence of the normalized successive enthalpy change on the guest-to-host molar ratio.

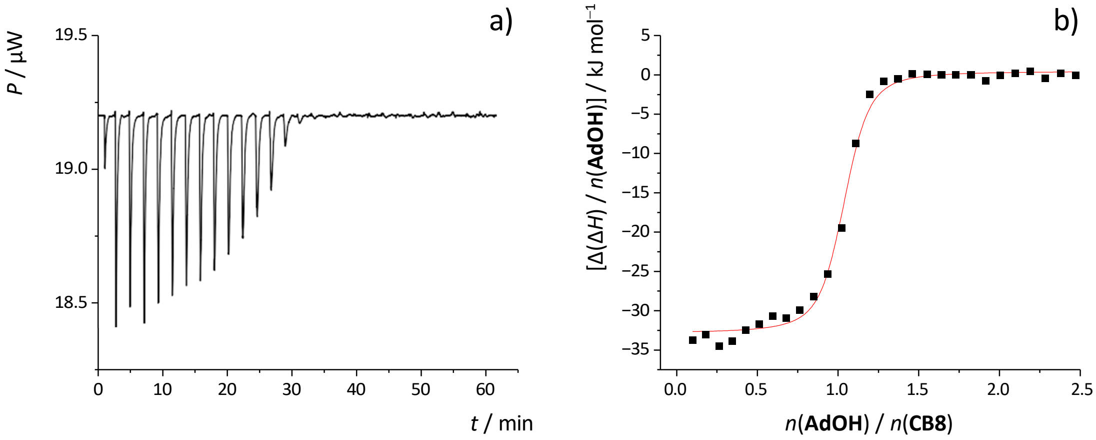

Figure S13. Microcalorimetric titration of **CB8** (c_0 = 3.21 × 10^{-5} mol dm^{-3}) with **AdOH** (c = 3.08 × 10^{-4} mol dm^{-3}) in D_2O at 298 K. a) Thermogram. b) Dependence of the normalized successive enthalpy change on the guest-to-host molar ratio. ■ experimental; – calculated.

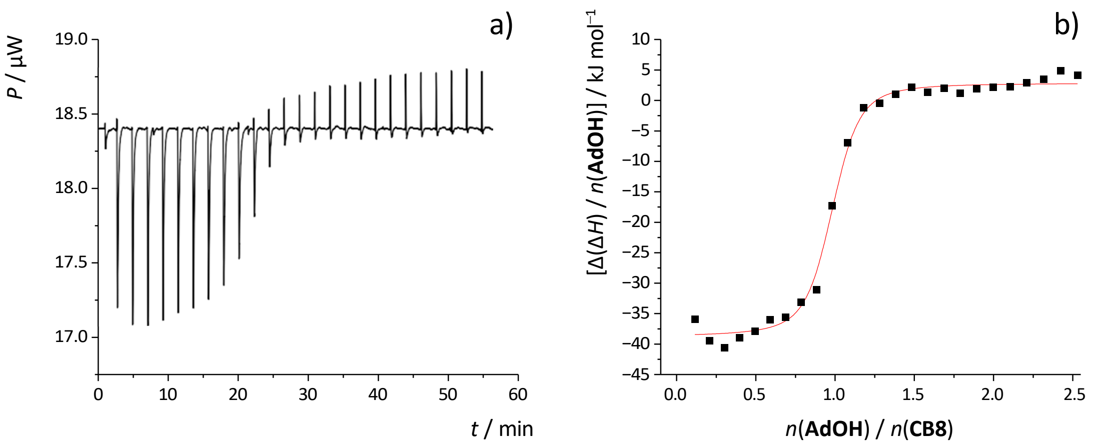

Figure S14. Microcalorimetric titration of **CB8** (c_0 = 3.21 × 10^{-5} mol dm^{-3}) with **AdOH** (c = 3.08 × 10^{-4} mol dm^{-3}) in D_2O at 318 K. a) Thermogram. b) Dependence of the normalized successive enthalpy change on the guest-to-host molar ratio. ■ experimental; – calculated.

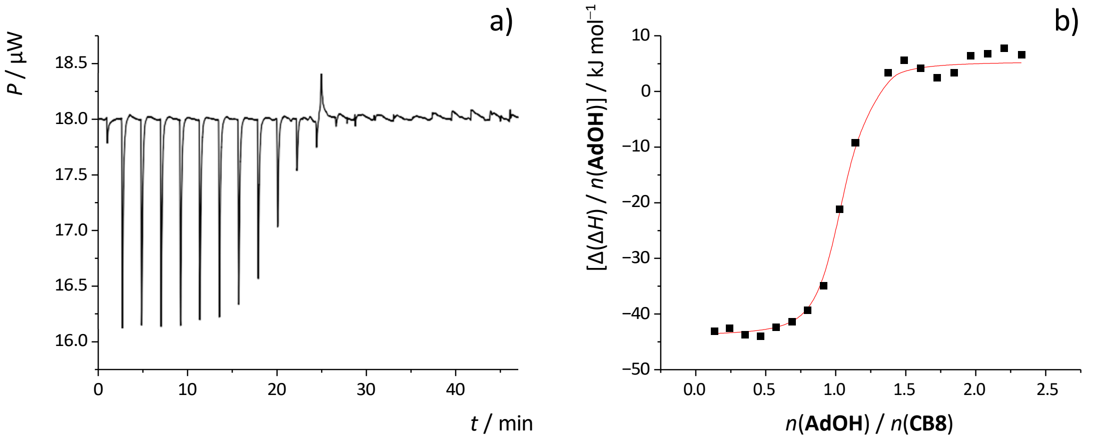

Figure S15. Microcalorimetric titration of **CB8** (c_0 = 3.21 × 10^{-5} mol dm^{-3}) with **AdOH** (c = 3.08 × 10^{-4} mol dm^{-3}) in D_2O at 338 K. a) Thermogram. b) Dependence of the normalized successive enthalpy change on the guest-to-host molar ratio. ■ experimental; – calculated.

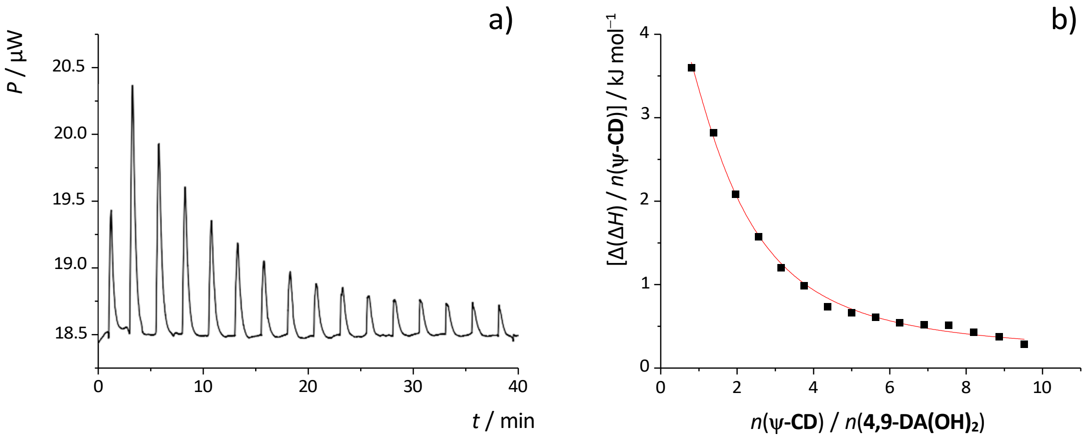

Figure S16. Microcalorimetric titration of **4,9-DA(OH)**$_2$ (c_0 = 1.22 × 10^{-4} mol dm^{-3}) with **γ-CD** (c = 5.65 × 10^{-3} mol dm^{-3}) in D_2O at 278 K. a) Thermogram. b) Dependence of the normalized successive enthalpy change on the host-to-guest molar ratio. ■ experimental; – calculated.

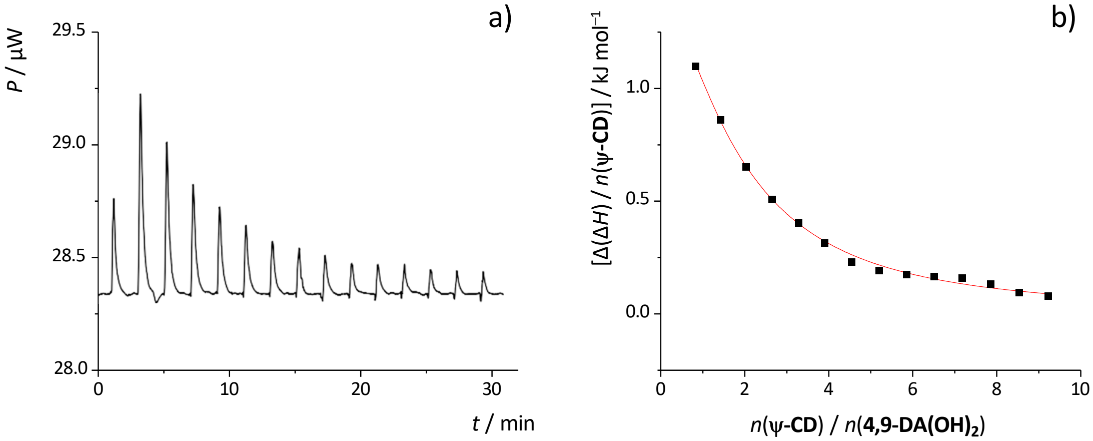

Figure S17. Microcalorimetric titration of **4,9-DA(OH)**$_2$ (c_0 = 1.22 × 10^{-4} mol dm^{-3}) with **γ-CD** (c = 5.65 × 10^{-3} mol dm^{-3}) in D_2O at 298 K. a) Thermogram. b) Dependence of the normalized successive enthalpy change on the host-to-guest molar ratio. ■ experimental; – calculated.

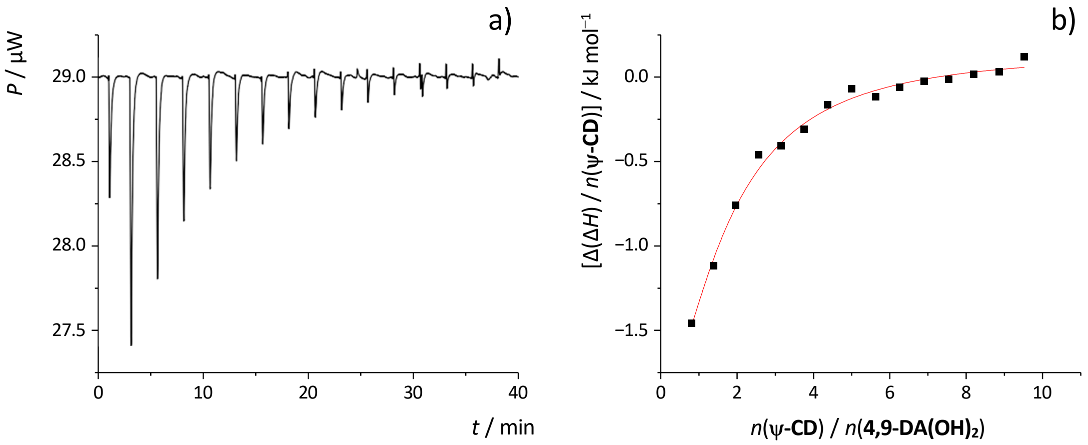

Figure S18. Microcalorimetric titration of **4,9-DA(OH)$_2$** (c_0 = 1.22 × 10^{-4} mol dm^{-3}) with **γ-CD** (c = 5.65 × 10^{-3} mol dm^{-3}) in D_2O at 318 K. a) Thermogram. b) Dependence of the normalized successive enthalpy change on the host-to-guest molar ratio. ■ experimental; − calculated.

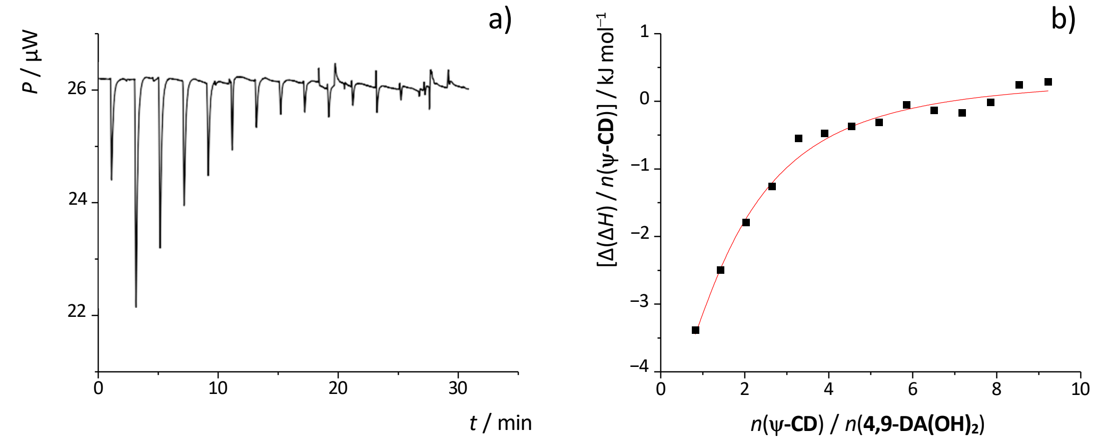

Figure S19. Microcalorimetric titration of **4,9-DA(OH)$_2$** (c_0 = 1.22 × 10^{-4} mol dm^{-3}) with **γ-CD** (c = 5.65 × 10^{-3} mol dm^{-3}) in D_2O at 338 K. a) Thermogram. b) Dependence of the normalized successive enthalpy change on the host-to-guest molar ratio. ■ experimental; − calculated.

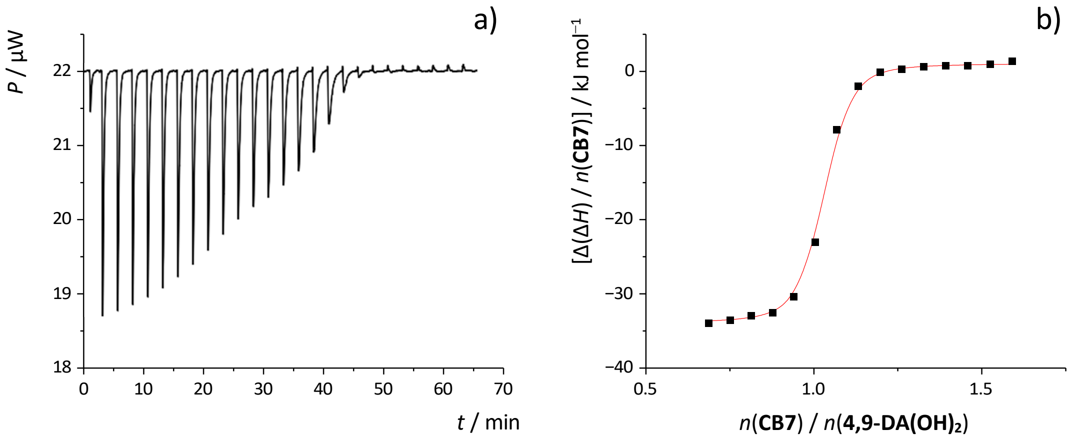

Figure S20. Microcalorimetric titration of **4,9-DA(OH)**$_2$ ($c_0 = 1.10 \times 10^{-4}$ mol dm^{-3}) with **CB7** ($c = 1.35 \times 10^{-3}$ mol dm^{-3}) in D_2O at 278 K. a) Thermogram. b) Dependence of the normalized successive enthalpy change on the host-to-guest molar ratio. ■ experimental; – calculated.

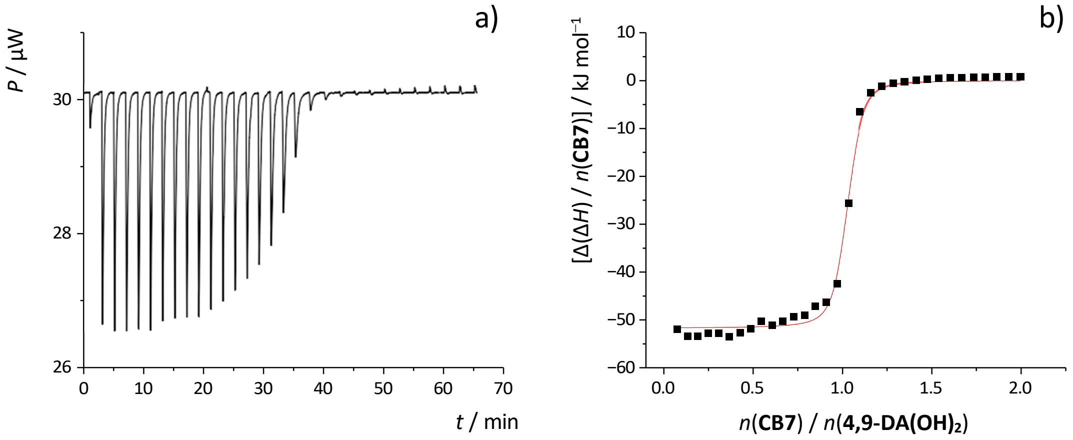

Figure S21. Microcalorimetric titration of **4,9-DA(OH)**$_2$ ($c_0 = 1.10 \times 10^{-4}$ mol dm^{-3}) with **CB7** ($c = 1.30 \times 10^{-3}$ mol dm^{-3}) in D_2O at 298 K. a) Thermogram. b) Dependence of the normalized successive enthalpy change on the host-to-guest molar ratio. ■ experimental; – calculated.

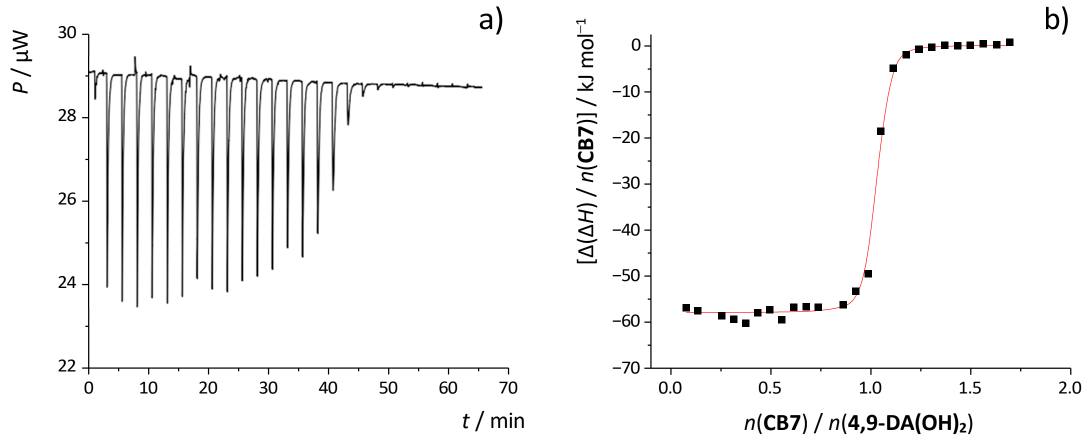

Figure S22. Microcalorimetric titration of **4,9-DA(OH)**$_2$ (c_0 = 1.10 × 10^{-4} mol dm^{-3}) with **CB7** (c = 1.31 × 10^{-3} mol dm^{-3}) in D_2O at 318 K. a) Thermogram. b) Dependence of the normalized successive enthalpy change on the host-to-guest molar ratio. ■ experimental; − calculated.

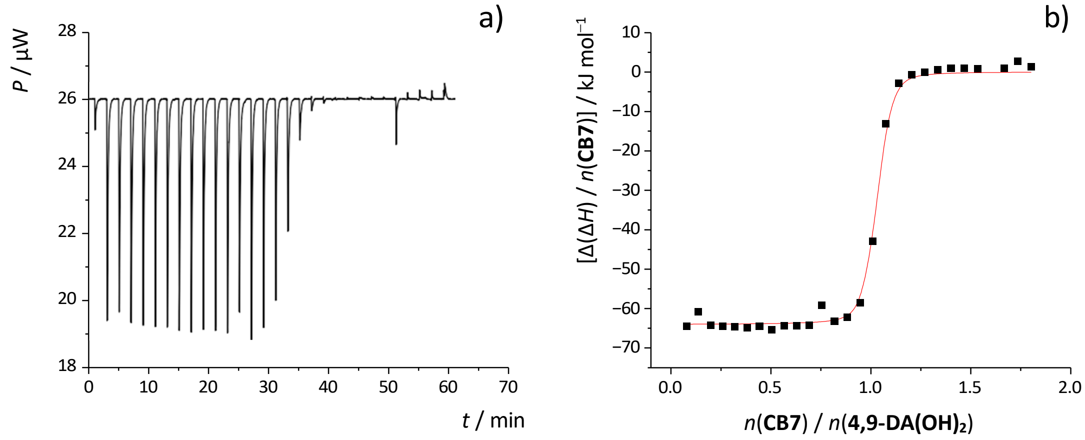

Figure S23. Microcalorimetric titration of **4,9-DA(OH)**$_2$ (c_0 = 1.10 × 10^{-4} mol dm^{-3}) with **CB7** (c = 1.39 × 10^{-3} mol dm^{-3}) in D_2O at 338 K. a) Thermogram. b) Dependence of the normalized successive enthalpy change on the host-to-guest molar ratio. ■ experimental; − calculated.

Temperature dependence of the standard thermodynamic parameters

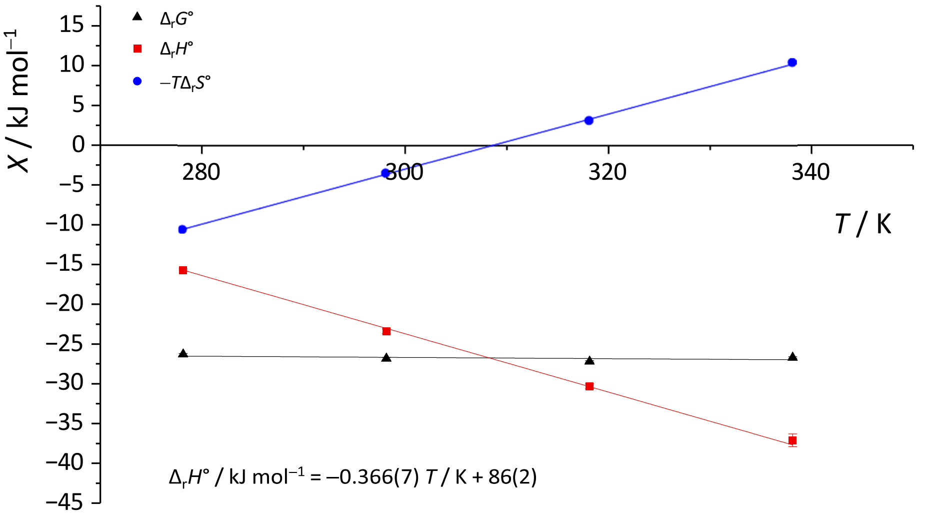

Figure S24. Temperature dependence of the standard thermodynamic parameters for the complexation of **AdOH** with **β-CD** in D_2O.

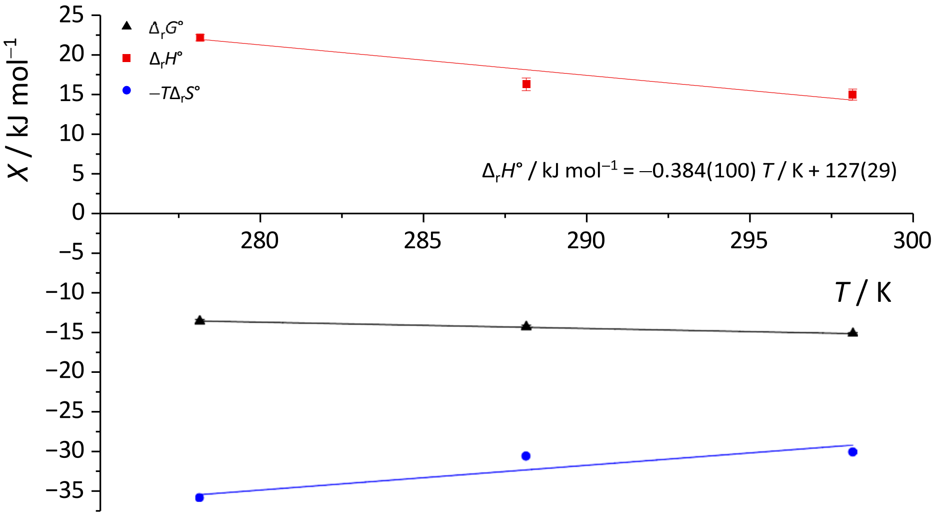

Figure S25. Temperature dependence of the standard thermodynamic parameters for the complexation of **AdOH** with **γ-CD** in D_2O.

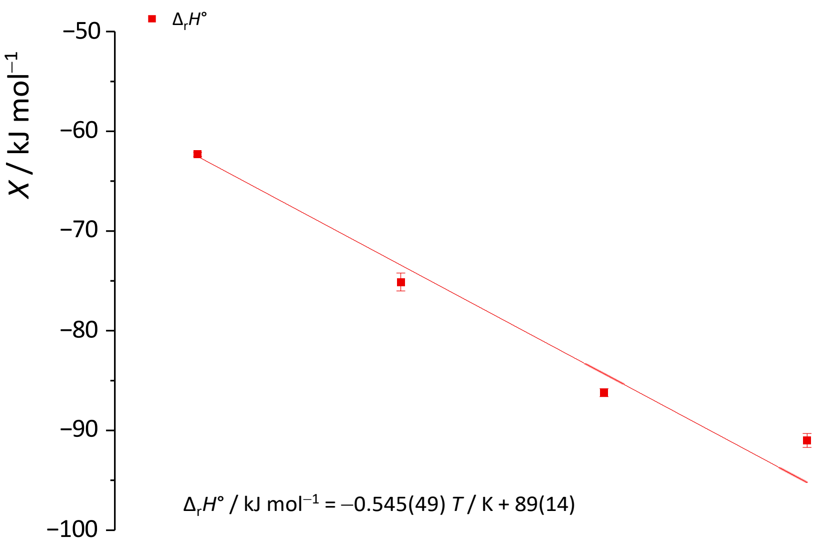

Figure S26. Temperature dependence of the standard reaction enthalpy for the complexation of **AdOH** with **CB7** in D_2O.

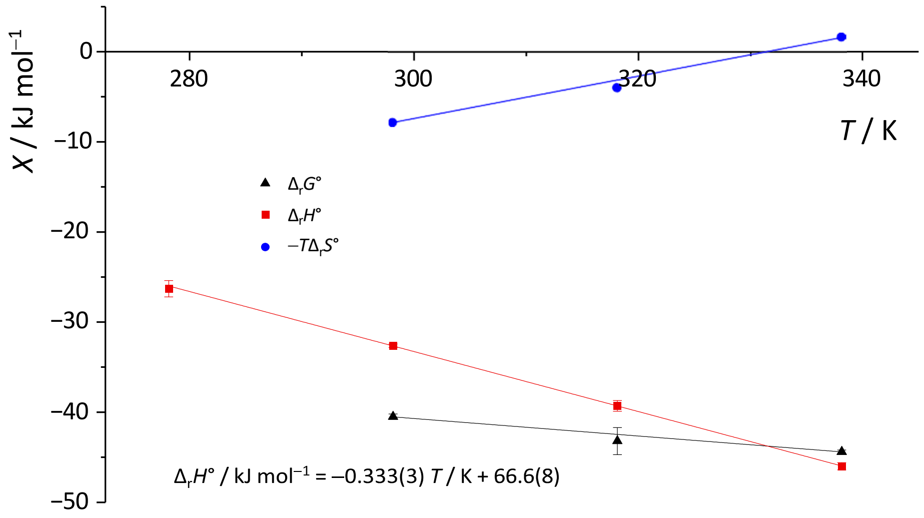

Figure S27. Temperature dependence of the standard thermodynamic parameters for the complexation of **AdOH** with **CB8** in D_2O.

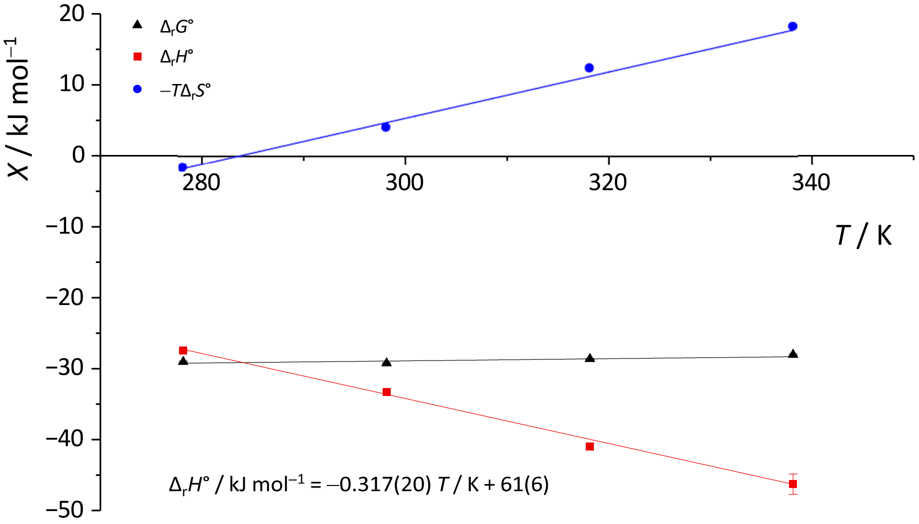

Figure S28. Temperature dependence of the standard thermodynamic parameters for the complexation of **4,9-DA(OH)**$_2$ with **β-CD** in D_2O.

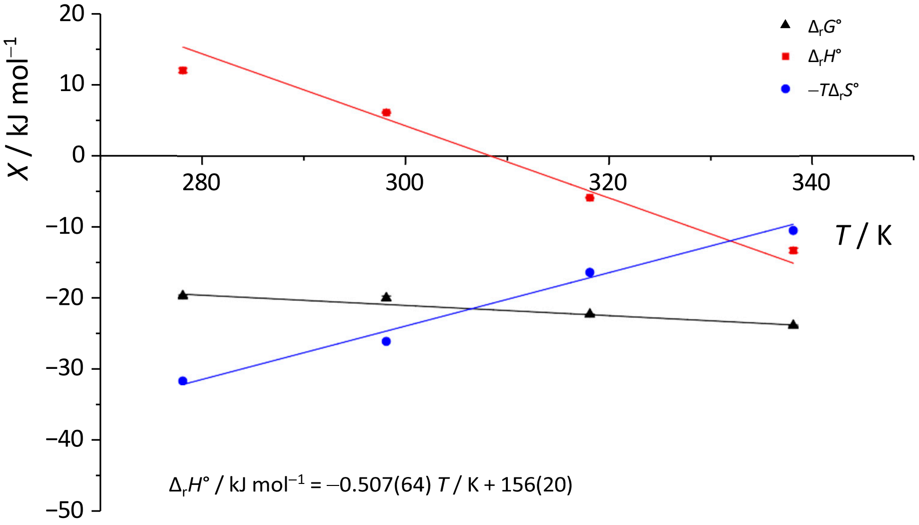

Figure S29. Temperature dependence of the standard thermodynamic parameters for the complexation of **4,9-DA(OH)**$_2$ with **γ-CD** in D_2O.

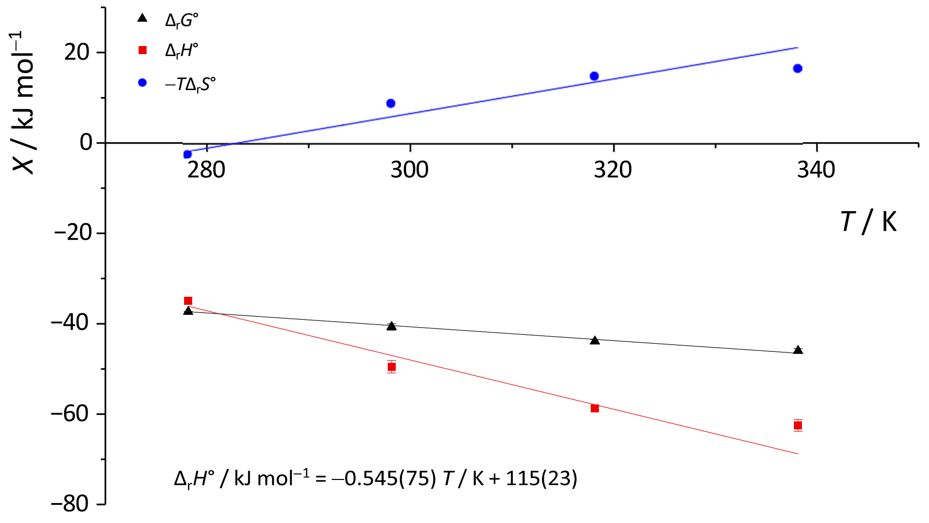

Figure S30. Temperature dependence of the standard thermodynamic parameters for the complexation of **4,9-DA(OH)**$_2$ with **CB7** in D_2O.

2. Statistical analysis

The following equations were used for error propagation analysis:

$$s(\Delta\log K^\circ) = [s^2(\log K^\circ(D_2O)) + s^2(\log K^\circ(H_2O))]^{1/2} \quad \text{(S1)}$$

$$s(\Delta\Delta_r G^\circ) = RT \ln 10\ s(\Delta\log K^\circ) \quad \text{(S2)}$$

$$s(\Delta\Delta_r H^\circ) = [s^2(\Delta_r H^\circ(D_2O)) + s^2(\Delta_r H^\circ(H_2O))]^{1/2} \quad \text{(S3)}$$

$$s(-T\Delta\Delta_r S^\circ) = [s^2(\Delta_r H^\circ(D_2O)) + s^2(\Delta_r H^\circ(H_2O)) + (RT \ln 10)^2 (s^2(\log K^\circ(D_2O)) + s^2(\log K^\circ(H_2O))) + 2RT \ln 10\ (\mathrm{Cov}(\log K^\circ, \Delta_r H^\circ \text{ in } D_2O) + \mathrm{Cov}(\log K^\circ, \Delta_r H^\circ \text{ in } H_2O))]^{1/2} \quad \text{(S4)}$$

$$\mathrm{Cov}(\log K^\circ, \Delta_r H^\circ) = [N(N-1)]^{-1} \Sigma_{i=1}^{N} (\log K^\circ_i - \langle\log K^\circ\rangle)(\Delta_r H^\circ_i - \langle\Delta_r H^\circ\rangle) \quad \text{(S5)}$$

where $\Delta\log K^\circ = \log K^\circ(D_2O) - \log K^\circ(H_2O)$ and $\Delta\Delta_r X^\circ = \Delta_r X^\circ(D_2O) - \Delta_r X^\circ(H_2O)$ ($X = G$, H, and S), s stands for the standard error of the difference between the means, $\log K^\circ_i$ and $\Delta_r H^\circ_i$ are the values obtained from the i-th titration, ⟨ ⟩ denotes the mean of these values over independent titrations (N = 3–5), each analyzed by a separate nonlinear regression, and Cov denotes the covariance of the two means, evaluated separately for each solvent.

Table S1. p-values for the two-sided Welch's t-test for the comparison of mean log $K°$, $\Delta_r H°$, and $-T\Delta_r S°$ determined in H_2O and D_2O. Values corresponding to statistically significant differences at the $\alpha = 0.05$ significance level are labeled with an asterisk.

Guest	Host	T / K	$p(\lvert t \rvert \geq \lvert t_{obs} \rvert)$		
			log $K°$	$\Delta_r H°$	$-T\Delta_r S°$
**AdOH**	**β-CD**	278	0.170	0.043 *	0.003 *
		298	0.097	<0.001 *	0.007 *
		338	0.036 *	0.506	0.834
	**γ-CD**	278	0.042 *	0.056 *	0.008 *
		288	0.325	0.709	0.416
		298	0.201	0.051	0.041 *
	**CB7**	278		0.010 *	
		298		0.229	
		318		0.004 *	
		338		0.329	
	**CB8**	278		0.427	
		298	0.032 *	0.058	0.007 *
		318	0.510	0.965	0.719
		338	0.358	0.465	0.376
**4,9-DA(OH)$_2$**	**β-CD**	278	0.126	0.879	0.470
		298	0.023 *	0.025 *	0.008 *
		318	0.185	0.172	0.276
		338	0.001 *	0.012 *	0.012 *
	**γ-CD**	278	0.026 *	0.140	0.012 *
		298	0.117	<0.001 *	<0.001 *
		318	0.033 *	0.043 *	0.039 *
		338	<0.001 *	<0.001 *	<0.001 *
	**CB7**	278	0.636	0.521	0.503
		298	0.769	0.106	0.164
		318	0.326	0.001 *	0.042 *
		338	0.222	0.302	0.235
